# Targeted deletion of a 170-kb cluster of LINE-1 repeats and implications for regional control

**DOI:** 10.1101/gr.221366.117

**Published:** 2018-03

**Authors:** Miguel L. Soares, Carol A. Edwards, Frances L. Dearden, Sacri R. Ferrón, Scott Curran, Jennifer A. Corish, Rebecca C. Rancourt, Sarah E. Allen, Marika Charalambous, Malcolm A. Ferguson-Smith, Willem Rens, David J. Adams, Anne C. Ferguson-Smith

**Affiliations:** 1 Department of Genetics, University of Cambridge, Cambridge CB2 3EH, United Kingdom;; 2 Departamento de Biomedicina, Unidade de Biologia Experimental, Faculdade de Medicina da Universidade do Porto, Porto; and i3S–Instituto de Investigação e Inovação em Saúde, Universidade do Porto, 4200-319 Porto, Portugal;; 3 Department of Veterinary Medicine, University of Cambridge, Cambridge CB3 0ES, United Kingdom;; 4 Wellcome Trust Sanger Institute, Wellcome Trust Genome Campus, Hinxton, Cambridge CB10 1SA, United Kingdom

## Abstract

Approximately half the mammalian genome is composed of repetitive sequences, and accumulating evidence suggests that some may have an impact on genome function. Here, we characterized a large array class of repeats of long-interspersed elements (LINE-1). Although widely distributed in mammals, locations of such arrays are species specific. Using targeted deletion, we asked whether a 170-kb LINE-1 array located at a mouse imprinted domain might function as a modulator of local transcriptional control. The LINE-1 array is lamina associated in differentiated ES cells consistent with its AT-richness, and although imprinting occurs both proximally and distally to the array, active LINE-1 transcripts within the tract are biallelically expressed. Upon deletion of the array, no perturbation of imprinting was observed, and abnormal phenotypes were not detected in maternal or paternal heterozygous or homozygous mutant mice. The array does not shield nonimprinted genes in the vicinity from local imprinting control. Reduced neural expression of protein-coding genes observed upon paternal transmission of the deletion is likely due to the removal of a brain-specific enhancer embedded within the LINE array. Our findings suggest that presence of a 170-kb LINE-1 array reflects the tolerance of the site for repeat insertion rather than an important genomic function in normal development.

Interspersed DNA elements are a hallmark of eukaryotic genomes with more than 40% of the human and mouse genomes consisting of repeats derived from transposable elements (International Human Genome Sequencing Consortium 2001; Mouse Genome Sequencing Consortium 2002). The most abundant class is the long interspersed elements (LINEs), which are autonomous retrotransposons and thus transpose via an RNA intermediate. This “copy and paste” mechanism results in mainly truncated copies of LINEs in the genome that are incapable of further retrotransposition. LINE-1 (L1) is the youngest of the four families and the major LINE family in eutherian and marsupial mammals. There are thought to be approximately 3000 active L1s in the mouse genome (Goodier et al. 2001) and around 100 in the human (Brouha et al. 2003). In the mouse there are three families of active L1s: L1md_A, L1Md_Gf, and L1Md_Tf (DeBerardinis et al. 1998; Goodier et al. 2001). Members of two of the active families, Gf and Tf, are known to be transcribed from the inactive X Chromosome in female mice, where they have been suggested to facilitate X Chromosome inactivation (XCI) in regions which would otherwise escape inactivation (Chow et al. 2010).

LINEs are found throughout the eukaryotes (Malik et al. 1999) and have played a major evolutionary role in sculpting the genomic landscape. They can generate structural variation through insertion-mediated deletion, in which the insertion of an L1 element can result in the deletion of the adjacent genomic sequence (Symer et al. 2002; Gilbert et al. 2002, 2005) and via nonallelic homologous recombination between L1s that can create insertions, deletions, and inversions (Han et al. 2008; Cordaux and Batzer 2009). Active L1s have been shown to be a major source of variation within the human population (Beck et al. 2010; Huang et al. 2010; Iskow et al. 2010) and can modulate gene expression, for example, by alternative splicing (Belancio et al. 2006), disrupting transcription (Han et al. 2004), and generating alternative promoters (Speek 2001).

Although some insertions may be beneficial, most insertions in and around genes are likely to be deleterious and thus attract epigenetic silencing to prevent retrotransposition. In the majority of somatic tissues, L1 activity is suppressed by DNA methylation at the promoter (Yoder et al. 1997). The importance of epigenetic silencing is evident in mice with deletion of the DNA methylation cofactor *Dnmt3l* which fail to methylate L1s in the germ line. In males, this results in pachytene arrest (Bourc'his and Bestor 2004). It is also evident that small RNAs play an important role in the suppression of L1 retrotransposition in the mammalian germline (Girard et al. 2006; Watanabe et al. 2006; Yang and Kazazian Jr. 2006; Kuramochi-Miyagawa et al. 2008). Finally the APOBEC3 family of RNA editing proteins have been shown to inhibit L1 activity in human cells (Bogerd et al. 2006; Chen et al. 2006; Muckenfuss et al. 2006; Richardson et al. 2014). However, despite all these defense mechanisms, L1s are still active in some tissues. Low-level L1 expression does occur in the male and female germlines (Branciforte and Martin 1994; Ostertag and Kazazian Jr. 2001); however, little retrotransposition occurs. Instead, most L1 retrotransposition occurs early in embryogenesis leading to somatic mosaicism (Kano et al. 2009). L1 retrotransposition has also been observed in neuronal progenitor cells (Muotri et al. 2005) and in the adult hippocampus (Coufal et al. 2009; Muotri et al. 2009). Estimates for the number of L1 insertions per neuron in human range from less than 0.1 to 80 (Coufal et al. 2009; Baillie et al. 2011; Evrony et al. 2012), with a recent study, using single-cell retrotransposon capture, estimating 13.7 insertions per hippocampal neuron (Upton et al. 2015).

The distribution of L1 repeats in the genome is not uniform: They tend to be found in AT-rich, gene-poor domains (International Human Genome Sequencing Consortium 2001), and they have been shown to be enriched close to monoallelically expressed genes such as olfactory receptor genes (Allen et al. 2003). In human they are almost completely absent from HOX clusters, yet there is a region on the X Chromosome spanning ∼100 kb that contains 89% LINEs (International Human Genome Sequencing Consortium 2001). It is not known whether such dense arrays of L1 repeats perform specific functions such as acting as boundary elements or if they represent repeat “graveyards,” accumulating only in regions where they can be tolerated in the genome. Here, we have identified 66 L1-dense regions of >100 kb within the mouse genome. One of the identified regions is rodent specific and lies within the proximal end of the mouse *Dlk1-Dio3* imprinted domain. Because LINEs have previously been shown to be enriched at imprinted regions (Allen et al. 2003), we have deleted this region and asked whether it is a “graveyard” for repeats or if it may have evolved a novel function within the rodent lineage. Here, we assess the consequences of the deletion on regional control of gene expression and imprinting.

## Results

### Large L1 arrays are associated with tandem gene clusters in the mammalian genome

**Figure 1.**
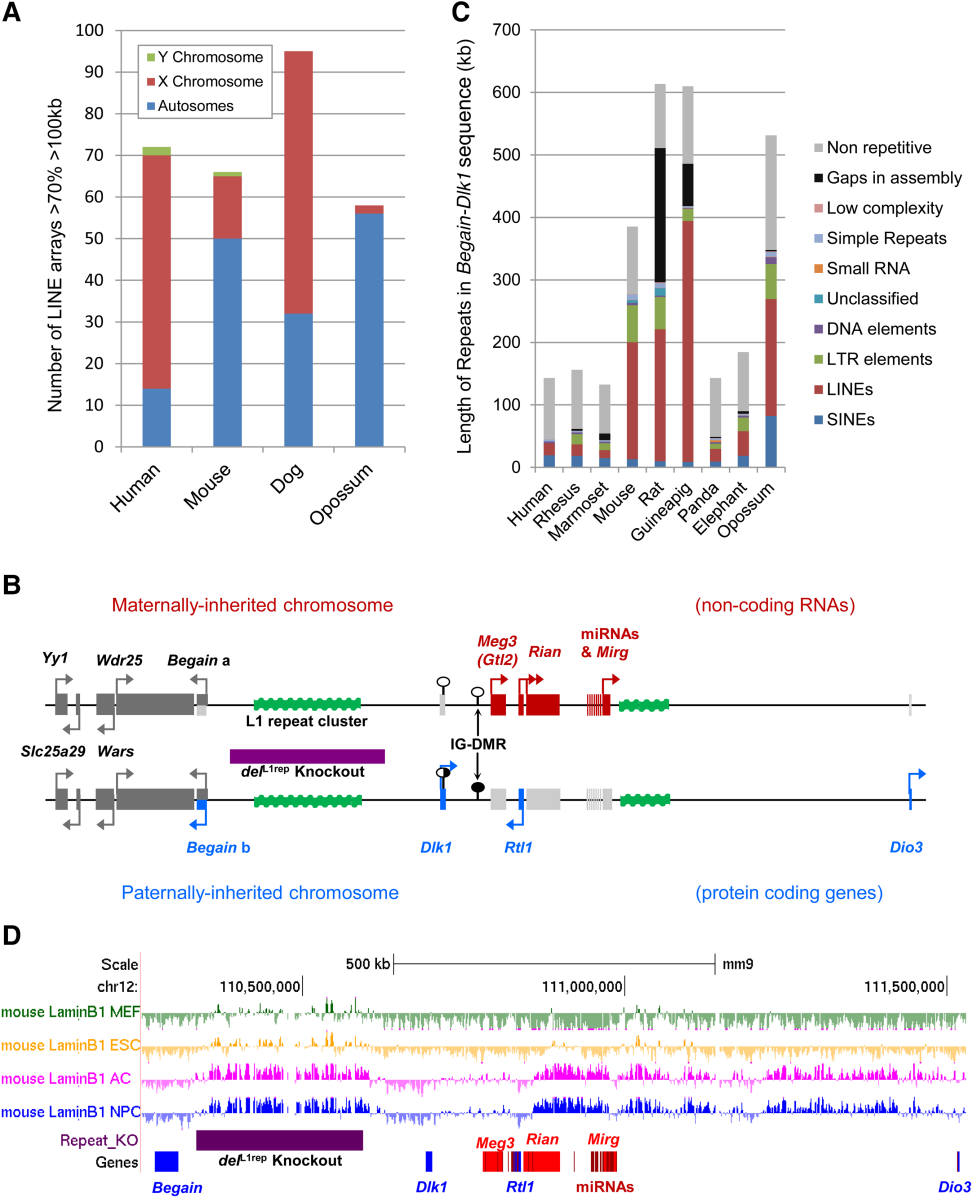
Distribution of LINE-rich arrays in mammalian genomes and identification of a large, intergenic LINE-rich repeat array in the mouse *Dlk1-Dio3* imprinted gene cluster. (*A*) Chromosomal distribution of LINE-rich arrays of >70% LINE over >100 kb in the human, mouse, dog, and opossum genomes. In the mouse genome, LINE-rich arrays of >70% LINE extending over 100 kb are associated with vomeronasal and olfactory receptor genes. (*B*) The LINE-rich repeat cluster (green) is flanked by the *Dlk1*-*Dio3* imprinted domain. Maternally expressed noncoding transcripts are shown in red, and paternally expressed protein coding genes are shown in blue. On the maternally inherited chromosome, noncoding RNAs *Meg3* (*Gtl2*), *Rtl1 anti-sense* (*AS*), *Rian*, and microRNA-containing *Mirg* (red) are transcribed, whereas the protein-coding genes (light gray) are repressed. The paternally inherited chromosome expresses protein-coding *Begain* variant 1b, *Dlk1*, *Rtl1*, and *Dio3* (blue), and silences the transcription of the noncoding RNAs (light gray). The imprinting control region (IG-DMR) and the *Dlk1* DMR are unmethylated on the maternally inherited chromosome (white circles), and hypermethylated/partially methylated, respectively, on the paternally inherited chromosome (black and shady gray circles). Biallelic genes are shown in dark gray, and arrows denote the direction of transcription. (*C*) The total repeat coverage in the *Begain*-*Dlk1* intergenic region in nine mammalian species illustrates the accumulation of LINE elements in the rodent lineage. Repeat content was ascertained using the RepeatMasker web server (Smit et al. 2015). (*D*) The mouse *Dlk1-Dio3* intergenic LINE array between *Begain* and *Dlk1* (purple bar) coincides with a facultative lamina-associated domain (fLAD) in astrocytes and neural precursor cells (Peric-Hupkes et al. 2010). Image taken from UCSC Genome Browser (Kent et al. 2002).

To determine where in the mouse genome stretches of dense LINE content reside, regions containing >70% LINEs of >100 kb length were cataloged. Sixty-six regions were identified: 50 were autosomal, with 15 on the X Chromosome and 1 on the Y Chromosome (Fig. 1A; Supplemental Table S1). Each region was then analyzed for gene content: 38 regions were intergenic, 4 were intragenic (within known transcripts), 11 were gene-flanking, and 13 were found both within and flanking genes (Supplemental Fig. S1A; Supplemental Table S1). Thirty-four of the LINE-rich regions contain genes; of these, 20 contain at least one gene belonging to a tandemly repeated gene cluster. These tracts of high LINE content are enriched for tandemly repeated olfactory and vomeronasal receptor genes (seven and eight regions, respectively; *P* < 0.01 when compared with 500 randomly generated genomic regions by binomial test). High LINE repeat content has previously been shown to be associated with random monoallelic genes such as the olfactory and vomeronasal receptors (Allen et al. 2003). Possible reasons for this association include a L1-mediated duplication mechanism in the evolution of these clusters (Younger et al. 2001) or a regulatory or structural role for L1s in monoallelic expression (Allen et al. 2003).

Because imprinted regions have previously been reported to be enriched for L1 repeats (Hutter et al. 2010) and three of the regions we identified reside in or flank imprinted domains (two in the *Snrpn* cluster and one in the *Dlk1-Dio3* region), we assessed whether there was enrichment for large arrays of LINE elements. For this analysis, LINE arrays were defined as regions of >50 kb in length with >40% of LINE content, which is double the mouse genome average (∼20%). Twenty-one imprinted domains and 1 Mb of flanking sequence on either side were assessed for LINE arrays, but no significant enrichment was found either within or surrounding imprinted gene clusters (Supplemental Fig. S1B).

When the lineage specificity was ascertained for all clustered and unclustered L1 repeats in the mouse genome, the ratio between the number within Eutherian, Rodent, Muridae, and *Mus* elements is approximately 1:1:1:1 (the number of L1s in each lineage = 203,859; 190,500; 216,000, and 262,981, respectively). However, in the LINE arrays, the numbers are skewed toward the Muridae and *Mus* specific elements with total numbers of elements within all 66 regions being 372, 747, 2004, and 2800, respectively. These data indicate that the accumulation of L1s in these particular regions is Muridae specific. To ascertain if this is the case, similar LINE-rich arrays were identified in three other species: human, dog, and opossum. Seventy-two regions of >70% LINEs extending over 100 kb were identified in the human genome (Fig. 1A), the majority of which (56) are located on the X Chromosome. Only one autosomal region is conserved between mouse and human: that containing the cytochrome gene cluster. In the dog, 95 regions were identified: 32 autosomal and 63 on the X Chromosome. Eight of the 32 autosomal regions contain olfactory receptor genes. Of the 58 arrays identified in the opossum genome, only two are located on the X. This is in agreement with previous observations that the opossum X is depleted for LINEs (Mikkelsen et al. 2007).

### Characterization of LINE-1 repeat array in the *Dlk1*-*Dio3* imprinted domain

We identified 40 intergenic LINE-rich arrays in mouse. Of these, only one is entirely located between two genes falling within a known coordinately regulated gene cluster, hence allowing this region to serve as a paradigm for functional analysis. This is the 170-kb array located between the *Begain* and *Dlk1* genes in the *Dlk1-Dio3* imprinted domain. This domain encompasses >1 Mb on mouse Chromosome 12 and contains paternally expressed protein-coding genes (*Dlk1*, *Rtl1*, *Dio3*, and *Begain* transcript b) and maternally expressed noncoding regulatory RNA transcripts (*Meg3* [also known as *Gtl2*], *Rtl1as*, and clusters of snoRNAs and miRNAs) (Fig. 1B). Imprinting is regulated via a long-range *cis*-acting mechanism controlled by a germ line–derived, intergenic, paternally methylated differentially methylated region (IG-DMR) located between *Dlk1* and *Meg3* (*Gtl2*) (Lin et al. 2003).

In order to assess the evolutionary context of this region in mammals, comparative sequence analysis was performed between the orthologous domains (start of *Begain* to the start of *Dlk1*) in nine mammalian species (Fig. 1C; Supplemental Table S2). In rodents, this region is almost three times as large as those of most nonrodent eutherians, in which the size is similar. The region in the rat and guinea pig is even more expanded than in the mouse (385,493 bp); however, there are a number of gaps in these reference genomes, and their true length is not known. The repeat content for the regions delineated by *Begain* and *Dlk1* was ascertained using RepeatMasker (Fig. 1C; Supplemental Table S2; Smit et al. 2015). All three rodent species show an increase in L1 content compared with other mammals. Further analyses demonstrate that the expansion seen in the mouse domain is due to the accumulation of L1 elements within a small interval (Supplemental Fig. S2A). The closest common evolutionary conserved regions (ECRs), reported in the UCSC Genome Browser, that span the 170-kb repeat array in the mouse are 226,694 bp apart, whereas in human, the distance separating these ECRs is only 2270 bp (Kent et al. 2002; Karolchik et al. 2004). A similar insertion is also present in the rat and guinea pig.

The ∼227 kb mouse insertion contains 92.0% interspersed repeats, the majority of which are L1 elements (67.8%). Lineage-specificity, membership of different families, and their age and ability to transpose were ascertained for all 157 L1 repeats identified within the insertion (Supplemental Table S3). The L1Base 2 database (Penzkofer et al. 2016) and the L1Xplorer program (Penzkofer et al. 2005) were then used to identify full-length L1s in the insertion. Nine putative full-length L1s were identified in the interval in L1Base 2 and one further full-length element by L1Xplorer (Supplemental Fig. S3; Supplemental Table S4), but none were found to contain intact open reading frames. Both Orf1 and Orf2 of all these elements have gaps, frameshifts, or stop codons, and most have mutated poly(A) signals (Supplemental Table S4). Together these data indicate the elements in this region cannot autonomously retrotranspose. Nonetheless, it is conceivable that these elements could still produce RNA transcripts (see below).

### The LINE-1-rich repeat array bordering the *Dlk1-Dio3* imprinted cluster is nuclear lamina associated

LINE-1s have been shown to be enriched in nuclear lamina-associated domains (LADs) (Meuleman et al. 2013). Analysis of publicly available DamID profiles of LaminB association data confirms that the 227 kb LINE-rich insertion upstream of *Dlk1* overlaps almost perfectly with a LAD in ES cell–derived neural precursor cells and astrocytes (Fig. 1D; Peric-Hupkes et al. 2010). This is a facultative LAD (fLAD) that is not present in the progenitor ES cell line or in mouse 3T3 embryonic fibroblasts. A further fLAD is present at the distal end of the imprinted domain running from the maternally expressed *Rian* gene to the paternally expressed *Dio3*. To determine whether association with LADs is a feature of all LINE arrays, the previously identified 66 regions of >70% LINE extending over 100 kb were intersected with LADs from the four cell lines, with a minimum of 10 kb overlap. Sixty-three of the 66 high-density L1 regions were found to overlap with a LAD in at least one of the cell lines, and 39 regions were constitutive LADs (cLAD) in all four lines (Supplemental Table S1).

### Deletion of 260 kb of the *Begain-Dlk1* LINE-1 repeat array

**Figure 2.**
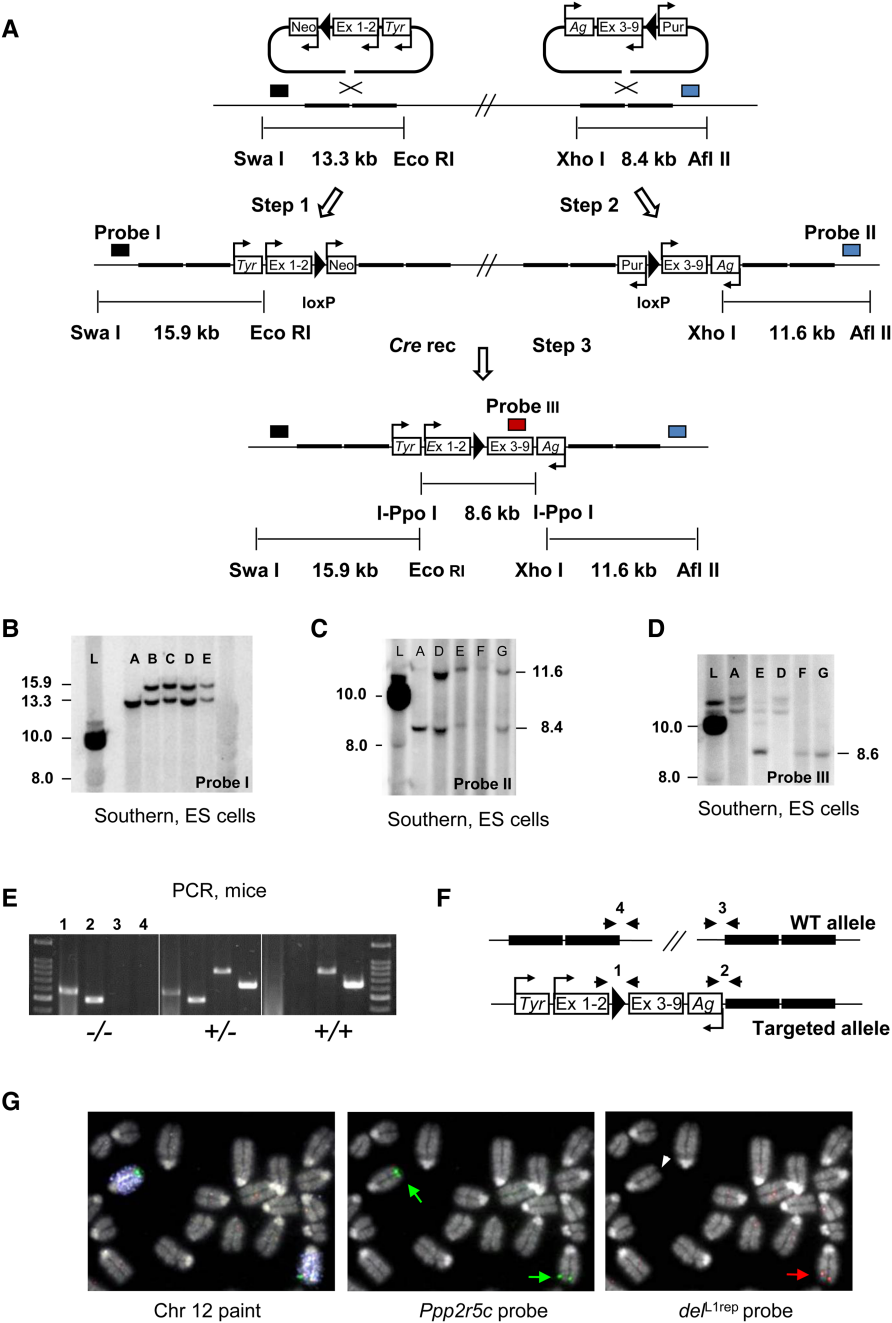
Targeting of 260 kb encompassing the LINE-rich repeat array: molecular and cytogenetic characterization of the *del*^L1rep^ allele. (*A*) Complementary MICER vectors (Adams et al. 2004) were consecutively integrated into the genome by homologous recombination in embryonic stem (ES) cells. Ex 1–2 and ex 3–9 correspond to the split *Hprt* minigene exons in the 5′ and 3′ vectors, respectively. After expression of Cre, the *loxP* elements recombine excising the intervening sequence and reconstituting the *Hprt* mini-gene, which results in resistance to hypoxanthine/aminopterin/thymidine (hat). Mini-genes for the coat color markers agouti (Ag) and tyrosinase (Tyr) are shown. The location and direction of promoter elements is indicated by arrows. Probes used for Southern hybridizations are indicated by boxes. Positions of restriction sites and the sizes of WT and targeted alleles are also shown. (*B*–*D*) Southern hybridization blots of ES cells at Step 1 (*B*), Step 2 (*C*), and Step 3 (*D*). (L) ladder. (*A*) wild type; (*B*,*C*) 5′ vector-targeted; (*D*) double-targeted (5′ and 3′ vectors). (*E*–*G*) Distinct *del*^L1rep^ (Cre-recombined) clones. (*E*) PCR genotyping of tail DNA of representative animals. (*F*) Schematic illustration of the respective assays. Primers pairs (1–4) used for screening are indicated by arrowheads. (*G*) Representative images of DNA FISH on metaphase spreads of mutant ES cells with BAC probes complementary to sequence within (*del*^L1rep^) and downstream (*Ppp2r5c*) from the deleted interval. The *Ppp2r5c* region (1 Mb downstream from the deletion) is identified in both Chromosome 12 homologs (green arrows), whereas the repeat region is seen in one homolog (red arrow) and is absent from the other (white arrowhead).

A large intergenic repeat cluster containing tandem repeat and retroelements has previously been reported between the *Igf2/H19* and *Kcnq1* imprinted domains; it has been proposed that this cluster may act as a target for epigenetic modifications that regulate imprinting (Shirohzu et al. 2004). To investigate whether the recently evolved, long, dense, lamina-associated LINE-1 array in the *Dlk1/Dio3* domain might have an impact upon imprinting and gene regulation, it was subjected to targeted deletion by homologous recombination in embryonic stem (ES) cells using vectors from the Mutagenic Insertion and Chromosome Engineering Resource (MICER) (Adams et al. 2004). The targeted interval (Chr 12: 109096083–109354908) is located 27.8 kb downstream from *Begain*, at its 5′ end and 98.3 kb upstream of *Dlk1* at its 3′ end and encompasses the entire 170-kb L1 array identified above (Fig. 1B). *Cre*-mediated recombination in ES cells produced a deletion of ∼260 kb of genomic sequence (Fig. 2A–F), which was confirmed cytogenetically by FISH (Fig. 2G). Chimeric mice bearing the deletion were generated and germ line transmission obtained: A mutant line (termed *del*^L1rep^) was then established on a C57BL/6J background.

Both heterozygous (*del*^L1rep^/+ and +/*del*^L1rep^) and homozygous animals were viable and appeared phenotypically normal. No deviations from the expected Mendelian ratio were observed in any of the crosses, and the average litter size and number of litters from each cross agrees with published data for C57BL/6J (Verley et al. 1967), showing uncompromised viability and reproductive fitness (Supplemental Table S5). Postnatal survival rates measured for periods of up to 52 wk revealed no differences between *del*^L1rep^ mutants and wild-type animals (Supplemental Table S5). There were also no significant differences in postnatal growth between wild types and *del*^L1rep^ mutants (Supplemental Fig. S4).

### LINE-1 elements from within the deleted repeat interval are transcribed biallelically

To assess LINE-1 transcription from within the repeat interval, two LINEs were shown to amplify from wild-type genomic DNA but not *del*^L1rep^ homozygous mutants, indicating the amplicons were specific to the deleted region (Supplemental Fig. S5A, left). These two amplicons were in the 3′ UTR of a full-length Lx2 LINE (L1Base 2 ID 11235) (Supplemental Fig. S3) and the 5′ UTR of an L1Md_F2 element (L1Base 2 ID 11087) (Supplemental Fig. S3). Transcription was assessed by RT-PCR in fetal brain and liver, and in adult brain. A Lx2-3′ UTR (11235) transcript was present in all three tissues assayed, and L1Md_F2-5′ UTR (11087) was transcribed in fetal and adult brain only (Supplemental Fig. S5A, right). Despite being full length, the open reading frames for these elements are not intact; thus, the transcripts do not code for functional L1 proteins, and the RNA transcribed has unknown relevance.

The allelic expression of L1 transcripts within the cluster was ascertained using fetal brain cDNA from embryos with uniparental duplications of Chromosome 12 (matUPD12 and patUPD12) (Georgiades et al. 2000). Amplification of the two LINE elements was observed both in mat and patUPD12 cDNA, indicating that the transcripts are not imprinted in fetal brain (Supplemental Fig. S5B) despite the imprinting of genes on either side of the array in this tissue. We cannot exclude that these L1 elements may be imprinted elsewhere in the body or in specific areas in the brain.

### Epigenetic control in the region is not affected by the absence of the LINE-rich array

To see if the LINE-rich array contributes to a landscape influencing the regulation of imprinted gene expression, we next examined the impact of *del*^L1rep^ on imprinting at the *Dlk1*-*Dio3* locus. Wild-type and heterozygous embryos with expressed polymorphisms on Chromosome 12 were obtained from reciprocal crosses between *del*^L1rep^ heterozygous mutants and *Mus musculus castaneus* (Cas) or *Mus musculus molossinus* 12 (Mol 12), a congenic mouse line derived in our laboratory harboring a *Mus molossinus Dlk1-Dio3* domain in a C57BL/6J background (Lin et al., 2003). Allele-specific expression of *Begain* b, *Dlk1*, *Meg3* (*Gtl2*), *Rtl1as*, and *Dio3* was ascertained by quantitative RT-PCR and pyrosequencing in fetal brain, liver, and placenta (*Begain* b and *Rtl1as* were only assessed in brain). All five transcripts were confirmed to be imprinted, retaining their original parent-of-origin specific expression (Supplemental Fig. S6). We also analyzed the allelic expression of *Wars*, *Wdr25*, and *Begain* a in the same tissues; each maintained their biallelic status (Supplemental Fig. S7). Hence deletion of the 170-kb L1 repeat cluster has no effect on imprinting at the region.

The allele-specific expression of all genes of the *Dlk1*-*Dio3* imprinted cluster and that of *Begain* variant b is controlled by the methylation status of the IG-DMR. This region is unmethylated on the maternal allele, where the protein coding genes are repressed and the noncoding transcripts are expressed; it is hypermethylated on the paternally inherited chromosome resulting in the expression of the protein coding genes and the repression of the noncoding transcripts. The mechanism behind this regulatory action is unknown, but it results in the simultaneous and coordinated expression control of genes spread over 1249 kb. Methylation of the IG-DMR—assessed by pyrosequencing—was unchanged in *del*^L1rep^ homozygotes, and maternal and paternal heterozygotes compared to wild-type samples (Supplemental Fig. S8). Together, these results indicate that the LINE-rich repetitive DNA tract is not involved in the establishment or maintenance of imprinting within the domain, nor is it required to shield the neighboring biallelic genes from the long-range epigenetic control exerted by the IG-DMR. The IG-DMR, however, appears to be unable to influence the allelic activity of the L1 transcripts within the cluster.

### Paternal transmission of *del*^L1rep^ results in temporal- and tissue-specific disruption of expression levels on the paternal chromosome through loss of an enhancer

**Figure 3.**
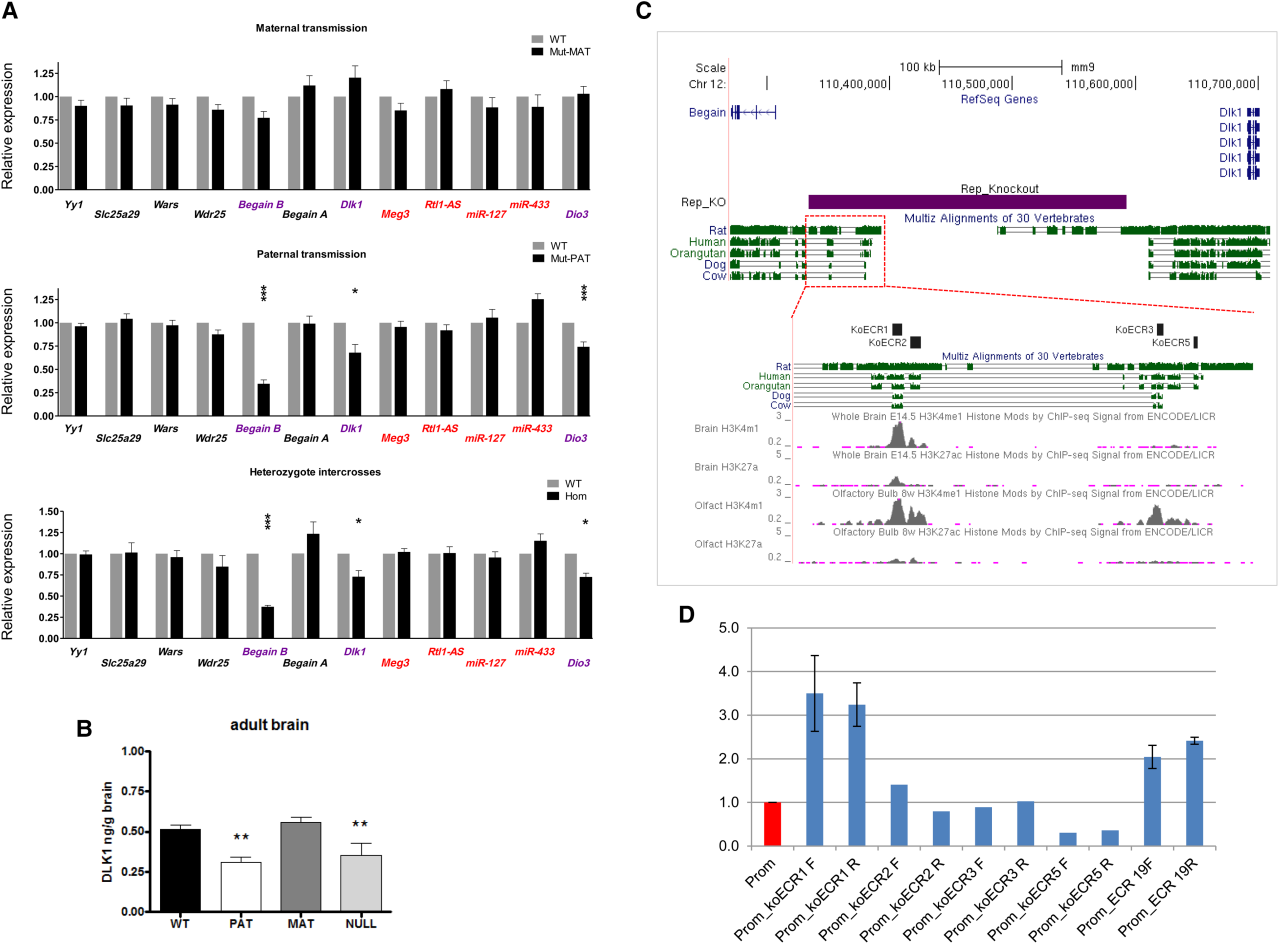
Paternal transmission of *del*^L1rep^ results in temporal- and tissue-specific disruption of imprinted gene expression on the paternal chromosome. (*A*) Relative expression of five biallelic genes closest to the deleted interval (black) and of the transcripts within the neighboring imprinted cluster ([blue] paternally expressed; [red] maternally expressed) in fetal (E16.5) brain, as determined by RT-qPCR. (*Upper*) No significant differences in expression were observed upon maternal transmission of the *del*^L1rep^allele (*n* = 12 wt, 14 mut; four litters). (*Middle*) Paternal inheritance of the *del*^L1rep^allele elicited a significant down-regulation of all of the paternally expressed genes (*n* = 13 wt, 13 mut; four litters). (*Lower*) The effect of *del*^L1rep^on the expression of *Begain* b, *Dlk1*, and *Dio3* was closely recapitulated in homozygous mutants (*n* = 12 wt, 13 mut; six litters). Maternally expressed miR-127 and miR-433 are part of larger transcripts (*Rtl1-AS* and *Mirg*, respectively) but have their own promoters and can be independently transcribed (Song and Wang 2008). Data were normalized to *Gap3dh* expression and are shown relative to WT controls (=1). (*) *P* < 0.05; (***) *P* < 0.001 by two-tailed Student's *t*-test. (*B*) DLK1 ELISA performed on wild type (WT), paternal transmission of *del*^L1rep^ (PAT), maternal transmission of *del*^L1rep^ (MAT), and homozygous adult brains (NULL). DLK1 protein levels in PAT and NULL are significantly lower than wild type: (**) *P* < 0.01 by ANOVA with Bonferroni's post hoc test. Error bars indicate SEM. (*C*) The *del*^L1rep^ region contains two evolutionary conserved regions (ECRs) as identified from the UCSC conservation track. ENCODE data for the enhancer-associated histone modifications H3K4me1 and H3K27ac in whole brain and olfactory bulb are also shown. The purple box denotes the deletion region; black boxes show the position of four conserved subregions that were used for luciferase assays. Image taken from UCSC Genome Browser (Kent et al. 2002). (*D*) koECRs 1, 2, 3, and 5 were cloned into pGL3-Promoter (Promega) in both orientations to test for enhancer activity. Constructs were transfected into E16.5 neurospheres. Graph shows the expression as measured in luminescence of luciferase relative to *Renilla* and normalized to the empty pGL3-Promoter vector (Prom). ECR19 was previously shown to have enhancer-like activity in ESCs and was shown to bind EP300 in brain (Visel et al. 2009). Statistics were calculated by the one-way ANOVA (and nonparametric) Friedman test to calculate the overall statistical significance (*P*-value), followed by Dunn's Multiple Comparison post-test to calculate the statistical significance (*P*-value) between specific samples. *P* < 0.05 was deemed as statistical significant. (Prom) pGL3-Promoter empty vector.

To further explore the effects of the repeat deletion at the molecular level, we first evaluated gene expression in wild-type and mutant E16.5 embryos and placentas. Twelve genes flanking the deletion were assayed by real-time quantitative PCR in embryonic brain, liver, and placenta, where expression of the Chromosome 12 imprinted genes is abundant. Maternal transmission of *del*^L1rep^ did not reveal detectable changes in gene expression in any of the three tissues (Fig. 3A, upper; Supplemental Fig. S9, middle). Normal levels of expression were also observed in liver and placenta in *del*^L1rep^ mutants after paternal transmission and in homozygous mutants from heterozygote intercrosses, when compared to wild-type littermates (Supplemental Fig. S9). Strikingly, we found that on paternal transmission, *del*^L1rep^ mutants exhibited significant down-regulation of the paternally expressed transcripts in the brain. Thus, the expression of *Begain* b, *Dlk1*, and *Dio3* in fetal brain was significantly decreased by 65%, 32%, and 26%, respectively (Fig. 3A, middle). This effect, which was specific to only the imprinted, protein-coding transcripts on the paternal chromosome, was recapitulated in homozygous mutants. *del*^L1rep^ homozygotes showed a comparable decrease in the expression of *Begain* b (63%), *Dlk1* (28%), and *Dio3* (28%) (Fig. 3A, bottom). Neither the biallelic nor the maternally expressed genes were found to have altered levels of expression in brain or in any of the tissues analyzed.

In the adult, in line with the results obtained with the embryo, maternal inheritance of the repeat deletion had no effect on the overall levels of gene expression in the mutant adult brain (Supplemental Fig. S10). In contrast, in paternal heterozygotes and homozygotes, *Dlk1* showed a significant decrease of 31%–34% (Supplemental Fig. S10). ELISA performed on wild-type, heterozygous, and null adult brains showed a >30% decrease in DLK1 protein levels in the null and paternally inherited deletion but no significant change upon maternal transmission (Fig. 3B; Supplemental Table S6). Taken together, our results show that paternal transmission of *del*^L1rep^ results in a tissue-specific reduction in expression of the imprinted genes on the paternally inherited chromosome.

Since functional elements within the genome evolve at slower rates than nonfunctional sequence, we scanned the repeat region for noncoding evolutionary conserved regions (ECRs). Genome alignments using the UCSC Genome Browser (Kent et al. 2002; Blanchette et al. 2004) identified two regions of conservation (Fig. 3C). These regions were then compared to whole-genome histone ENCODE ChIP-seq data (The ENCODE Project Consortium 2011) to determine if they overlapped established enhancer-associated histone signatures, such as H3 lysine 27 acetylation (H3K27ac) and H3 lysine 4 mono-methylation (H3K4me1), in neuronal lineage cells. One region, koECR1, was found to overlap with H3K4me1 in whole brain and olfactory bulb. KoECR2 and 3 also showed an H3K4me1 association in olfactory bulb (Fig. 3C).

These putative enhancers were tested for enhancer activity using in vitro luciferase reporter assays in neurosphere cells derived from the cortex of E16.5 brains. Enhancer-like activity (3.2- to 3.5-fold greater than empty vector) was observed from the koECR1 region in both orientations (Fig. 3D) consistent with positive control constructs generated using a known neural enhancer. The other three regions tested showed no enhancer-like activity in these cells, although they may have regulatory roles in other areas of the brain or tissues. However, no enhancer-like activity was observed from any of the regions in NIH-3T3 fibroblasts, mouse ES cells (E14Tg2a), or neural stem cells derived from 46C ES cell line (Supplemental Fig. S11; Ying et al. 2003). *Dlk1* is expressed in fetal neurospheres (Ferrón et al. 2011), whereas *Begain* is not, suggesting koECR1 may function to enhance *Dlk1* expression in these cells.

Together, our data indicate that the large dense array of LINE-1 elements residing within a coordinately controlled, epigenetically regulated imprinted domain, does not contribute to a landscape required for the genomic imprinting or transcriptional regulation of adjacent genes across a 1.25-Mb region.

## Discussion

### Dense arrays of LINEs accumulate in regions of the genome where they can be tolerated

Nearly half of the mammalian genome is composed of repetitive sequences, yet their influence on genome function is only now beginning to be considered, predominantly due to previous technical challenges associated with working with repetitive elements. Here, we characterized a class of large LINE repeat arrays and asked whether one such cluster of L1 repeats might function as a modulator of local control at a well-characterized imprinted cluster on mouse Chromosome 12.

It was previously suggested that coordinately regulated gene clusters are devoid of repeats because they are under the control of *cis*-acting elements that cannot tolerate their presence, for example, *Hox* clusters (International Human Genome Sequencing Consortium 2001; Simons et al. 2006). In agreement with this is the fact that the majority of the large LINE arrays identified were found in intergenic regions (38/66).

Dense LINE arrays were shown to be enriched in regions of tandemly duplicated olfactory and vomeronasal receptor genes in mice. The association between LINEs and random monoallelic genes, such as olfactory receptors, has been long established, and it is suggested that these LINEs may facilitate monoallelic expression (Allen et al. 2003). It has also been claimed that L1-mediated mechanisms may be responsible for the duplication of these genes (Younger et al. 2001). The large numbers of LINEs that flank these genes may be tolerated because they provide a mechanism by which the gene repertoire can be increased, allowing the animals to adapt more quickly to changing environments or indeed because they sit harmlessly outside regions required for the regulated allelic exclusion associated with such domains.

Similarly, imprinted gene clusters are coordinately regulated, reflecting both their *cis*-acting parental origin-specific imprinting control and their coregulation in common developmental or physiological pathways (Ferguson-Smith 2011; Charalambous et al. 2012). The existence of a large L1 repeat domain at the proximal end of the *Dlk1-Dio3* imprinted domain raised questions as to whether it was performing some sort of topological role, or alternatively that the region provided a safe locale for a “graveyard” for such elements.

We deleted the intergenic LINE-rich array in the mouse *Dlk1-Dio3* domain to ascertain the implications of the presence of a large repeat region on a large gene cluster, whose monoallelic expression is governed by a single *cis*-acting element. This repeat array was found to be rodent specific, and its existence within the *Dlk1-Dio3* domain indicates that the gene cluster is able to tolerate such an accumulation without any detrimental effects. Clearly, the *cis*-acting function of the IG-DMR is unaffected by the presence of a repeat insertion since imprinting in the domain occurs in eutherian mammals with and without the LINE array (Smit et al. 2005; Hagan et al. 2009). Moreover, deletion of the LINE-rich region does not alter the imprinting status of any of the genes in the region. One possible function of such LINE-rich regions in the genome could be as boundary elements between differentially regulated genomic regions. This is not the case for the murine *Dlk1-Dio3* LINE array since the IG-DMR is still able to exert its influence on the proximal *Begain* b gene to confer its paternal origin-specific expression in mouse despite the presence of the LINE-rich insertion.

LINE-1s have previously been found to be enriched in the regions surrounding imprinted and random monoallelic genes (Allen et al. 2003). This finding led to speculation that LINEs in autosomal monoallelically expressed regions were acting as “way stations” to aid the spreading of heterochromatin via a mechanism similar to that hypothesized for *Xist* in X inactivation at the time (Lyon 1998). However, this idea has been recently thrown into doubt because it is now believed that marsupials, who have LINE-poor X Chromosomes, also use a noncoding RNA (*Rsx*) to achieve XCI (Grant et al. 2012). In mice, the *Dlk1-Dio3* domain extends from *Begain* through to *Dio3* (Fig. 1B), and this is the same as in sheep (Smit et al. 2005), which do not contain a large LINE-1 cluster. The lack of conservation of this LINE-1 array indicates that it does not have a key role in conferring a particular chromatin state required for long-range control. Furthermore, the deletion of the mouse repeat has no effect on the imprinting status of any of the genes in the region or the methylation status of the IG-DMR, indicating that the repeat array has gained no role in the control of monoallelic gene expression in this imprinted cluster under normal developmental or physiological conditions.

### Gain of function in gene regulation and chromosome architecture

Inheritance of *del*^L1rep^ from either parent causes no gross phenotypic effects; however, there are changes to expression levels of paternally expressed genes within the region. These expression changes were only observed in embryonic and adult brain and suggest that any possible phenotype in the knockout mice may be behavioral. One likely explanation for reduction in paternally expressed transcripts upon paternal transmission is that brain-specific enhancers could have been removed along with the deletion. We identified a putative brain-specific enhancer on the basis of conservation and of an enhancer-associated epigenetic signature; moreover this element exhibited cell-type–specific enhancer activity in reporter assays in ex vivo neural cells. Alternatively, both LTRs and SINEs have previously been demonstrated to exhibit enhancer functions in some contexts (Hambor et al. 1993; Long et al. 1998), so it is also possible that the repeat elements themselves may have gained an enhancer function. This has previously been observed at the human lipoprotein, *Lp(a)* locus (Wade et al. 1997; Yang et al. 1998).

The *del*^L1rep^ mutant displays no obvious physical phenotype. However, as L1 expression in the region was only observed in brain and changes to expression levels of local imprinted genes was only observed in neural tissues, it would be interesting to see whether there may be behavioral phenotypes in mutant mice, including whether the mutant mice react differently in stress conditions.

DamID data indicate that the repeat array is nuclear lamina-associated in neural precursor cells (Peric-Hupkes et al. 2010), and that these LINEs may contribute to the tethering of particular genomic regions to the nuclear periphery. In some contexts, nuclear lamina association has previously been shown to lead to reduction in gene expression (Finlan et al. 2008). It has recently been shown that in ES cells, the maternally inherited *Dlk1-Dio3* chromosome, which is highly expressed for *Meg3* (*Gtl2*), is preferentially located internally in the nucleus, and the paternally inherited locus from which little expression is observed is located peripherally (Kota et al. 2014). Perturbation of maternal gene expression by knocking down expression from the IG-DMR resulted in more peripheral localization, indicating that lack of expression itself may target a region to the periphery (Kota et al. 2014). Here, we show that upon maternal transmission of *del*^L1rep^, no significant changes in *Meg3* (*Gtl2*) expression or any other genes in the domain was observed in any tissues analyzed. Furthermore, upon paternal transmission of *del*^L1rep^, the expression of both *Dlk1* and *Begain* b is actually reduced in the brain, indicating that the lamina association in the region is not repressing gene expression, again supporting a causal role for a putative neural enhancer within the deleted interval. It is also of note that the *Dlk1-Dio3* domain contains a second longer neuronal lineage LAD, suggesting that the region might remain tethered to the lamina even after deletion of the L1-rich array.

Together, our findings suggest that a 170-kb LINE-1 array, located between two paternally expressed imprinted genes within the *Dlk1-Dio3* imprinted domain, reflects the tolerance of the site for repeat insertion rather than an important genomic or epigenetic function.

## Methods

### In silico analysis

For the whole-genome analyses, the following genome builds were used: Mouse, mm10; Human, hg38; Dog, canFam3; Opossum, monDom5. Whole-genome repeat annotations were downloaded from RepeatMasker (Smit et al. 2015). These annotations were uploaded into the SeqMonk program (developed by Simon Andrews at the Babraham Institute, https://www.bioinformatics.babraham.ac.uk/projects/seqmonk/). The percentage coverage by LINEs was ascertained for running windows of 100 kb with 10-kb steps. Next for each domain, the percentage covered by >70% LINEs >100 kb was ascertained. The genes in each region were obtained using the UCSC Table Browser (Karolchik et al. 2004). The coordinates of known LADs were taken from Peric-Hupkes et al. (2010) and intersected with the 68 regions with >70% LINE >100 kb using the Join utility in Galaxy (Giardine et al. 2005; Blankenberg et al. 2010; Goecks et al. 2010) for a minimum of 10 kb overlap. For the imprinted region analysis, running windows of 50 kb with 10-kb steps were used and regions of >40% LINE >50 kb were identified. Random sequences were generated using BEDTools (Quinlan and Hall 2010). Wilcoxon matched pair test was performed using GraphPad Prism version 5.00 for Windows, GraphPad Software (https://www.graphpad.com).

For *Begain*-*Dlk1*, repeat analysis in genomic coordinates and sequence were taken from the UCSC Genome Browser (http://genome.ucsc.edu/) (Kent et al. 2002). Repeat content was ascertained using RepeatMasker Web Server (Smit et al. 2015). Information on the lineage specificity of the LINE repeats was obtained from RepeatMasker (http://www.repeatmasker.org/) (Smit et al. 2015) and Repbase (http://www.girinst.org/repbase/).

### Gene targeting in ES cells

The targeting vectors used to generate the allele with the deletion (*del*^L1rep^) were obtained from the Mutagenic Insertion and Chromosome Engineering Resource (MICER, http://www.sanger.ac.uk/resources/mouse/micer/) and have been described in detail previously (Adams et al. 2004). Ready-made MICER vectors MHPN49e02 (Chr 12: 110,325,911–110,334,290, NCBIM37) and MHPP67j17 (Chr 12: 110,593,112–110,597,765) were customized by the introduction of a I-PpoI restriction site in the *tyrosinase* 5′ flank region (878/880, MHPN) and in the vector backbone (6245/6249, MHPP). After digestion with PacI and SbfI, respectively, a double-stranded DNA oligonucleotide containing the I-PpoI restriction site with matching overhangs for each of the enzymes was ligated into the vectors by common procedures. I-PpoI (Promega) is a yeast-specific intron-encoded endonuclease from *Physarum polycephalum* that does not cut mammalian DNA or any of the commonly used plasmids or vectors.

Feeder-independent E14Tg2a ES cells cultured in KO-DMEM (Invitrogen) supplemented with 15% FBS (Hyclone), 1% beta-Mercaptoethanol, 1% L-glutamine/Pen-Strep solution, and ESGRO (LIF, Chemicon) supplement at 1000 units/mL, were electroporated with 50 µg of SmaI-linearized MHPN vector and selected with G418 (180 µg/mL) (Sigma). Targeting was determined by Southern blotting following digestion of genomic DNA with SwaI and EcoRI. Positive clones were subsequently electroporated with 50 µg of KpnI-linearized MHPP vector and selected with puromycin (3 µg/mL) (Sigma). Targeting was ascertained by Southern blotting following digestion of genomic DNA with XhoI and Afl II. Two double-targeted clones were transfected transiently with the Cre-expressing vector pOG231 (Addgene plasmid 17736) (O'Gorman et al. 1991) by electroporation and selected with hypoxanthine/aminopterin/thymidine (HAT) (Gibco). HAT-resistant colonies were genotyped by Southern blotting as described above and following double digestion with NdeI and I-PpoI. Successful deletion was confirmed by the presence of an 8.6-kb fragment resulting from the I-Ppo I digestion within the two neighboring targeting vectors, which demonstrated the removal of the intervening sequence. Euploidy was verified for five ES cell clones carrying the *del*^L1rep^ allele by chromosome counting of 50 metaphase spreads per clone.

### DNA fluorescence in situ hybridization (FISH)

Metaphase spreads of ES cells carrying the *del*^L1rep^ allele were hybridized with two BAC clones from the 129S7/AB2.2 BAC repository (Sanger Institute). bMQ-177c10 (110,338,144–110,464,879) is located within the deleted interval; bMQ-98C5 (111,624,966–111,780,688) partially overlaps *Ppp2r5c*.

Mouse metaphase preparations were prepared from primary fibroblast cultures, grown in Dulbecco's modification of minimal essential medium (Gibco), enriched with 10% fetal bovine serum (Gibco), penicillin (100 units/mL) and streptomycin (100 mg/mL). Chromosome preparations were made following standard procedures that included 1 h of colcemid treatment (0.05 mg/mL) followed by 8 min of hypotonic treatment in 0.075 M KCl and fixation in 3:1 methanol/glacial acetic acid. Ten microliter metaphase preparation was dropped onto glass slides followed by 10 µL 3:1 methanol:acetic acid fixative in a 52°C water bath to achieve sufficient spreading. To remove excess cytoplasm, slides were dehydrated in 100% ethanol for 5 min and treated with 0.0025% pepsin in 10 mM HCl for 2 min. Slides were then rinsed in 2× SSC twice for 5 min followed by a wash with deionized water. Slides were then passed through an ethanol series of 70%, 90%, and 100% ethanol. Slides were then air dried and baked for 1 h at 65°C. BAC DNA was purified using the Sigma PhasePrep BAC DNA Kit (NA0100-1KT) and labeled with biotin or dUTP-Cy3 by a standard Nick Translation procedure using 1 µg of purified BAC DNA. Chromosome 12 Paint (Cambio) and approximately 150 ng BAC probe and 1 µg Mouse Hybloc DNA (Applied Genetics Laboratory MHB-0.5) were made up to 12 µL with hybridization buffer (50% deionized formamide, 10% dextran sulfate, 2×SSC, 0.5 M phosphate buffer, pH 7.3, and 1×Denhardt's solution) and denatured for 10 min at 75°C and preannealed for 30 min at 37°C. Metaphases were denatured for 1 min 40 sec at 67°C in 70% formamide in 2×SSC. Immediately after denaturing, slides were placed in ice cold 70% ethanol for 4 min. The ethanol series was repeated. BAC probes were applied to the slide and covered with a coverslip, sealed with fixogum, and incubated for two nights at 37°C in a humid chamber. Post-hybridization washes were performed at 42°C in 50% formamide, 2×SSC for 5 min twice, 2×SSC for 5 min twice, and 4×T (0.05% Triton X-100 in 4×SSC) for 4 min. Biotin-labeled probes were visualized using 1:500 Streptavidin-Cy3 (GE Healthcare) which was applied to the slides in 4×T covered with parafilm and incubated for 30 min at 37°C. Slides were rinsed in 4×T for 3 min, twice. Excess fluid was removed, and slides were mounted in DAPI II and sealed with nail varnish. Images were captured using a wide-field Leica DMRXA fluorescence microscope and a ×100 oil immersion objective with a numerical aperture of 1.4.

### Generation of chimeric mice and germ line transmission of the *del*^L1rep^ allele

All experimental procedures with live animals were conducted in accordance with UK Government Home Office Licensing regulations. CD-1 embryos were recovered at the four-cell stage from the oviducts of fertilized CD-1 females and cultured in KSOM medium up to the uncompacted eight-cell stage. Clumps of 6–15 mutant ES cells were placed in purpose-built wells covered with ES cell medium, in close contact with host morulae, which were allowed to develop to the blastocyst stage. Blastocysts were implanted into the uteri of pseudopregnant CD-1 females and the born chimeric animals were readily identified by the coat color, due to the expression of the Agouti minigene in the white CD-1 background. Chimeric males were mated to albino C57BL/6 females (Charles River) for germ line transmission of the *del*^L1rep^ allele. Heterozygous mutant progeny were easily discriminated by the coat color (white-bellied chinchilla), the genotypes of which were confirmed by PCR and Southern blotting of genomic DNA isolated from tail biopsies at weaning (details below). A mutant line was then established by backcrossing the *del*^L1rep^ allele onto the C57BL/6J background over 10 generations.

### PCR and Southern blot genotyping

Genomic DNA isolated from tail biopsies by phenol/chloroform and from ear fragments with the Tissue-to-PCR kit (Fermentas) was PCR amplified using four sets of primers as follows: Pair 1, Fw CAAGCACTGGCTATGCATGT, Rv TGAACCCAGGAGGTTGAGAC, 60°C annealing, 40 cycles, 2 min 72°C extension; pair 2, Fw TTTAGAGCTTGACGGGGAAA, Rv CAAGAGCTCTGGGGTACTGG, 55°C annealing, 35 cycles, 30 sec 72°C extension; pair 3, Fw AGCGGATCCATCCTTTGAGT, Rv CAAGAGCTCTGGGGTACTGG, 65°C annealing, 34 cycles, 1 min 72°C extension; pair 4, Fw ACCAACCTCTCTGTGGCTGTG, Rv TCCACACGAGGACACCATGCC, 65°C annealing, 32 cycles, 30 sec 72°C extension. Primer pair 1 produces an amplicon of 589 bp spanning the recombination breakpoint on the mutant allele; pair 2 amplifies a fragment of 494 bp that spans the boundary between the MHPP backbone vector and the 3′ genomic arm on the mutant allele; pairs 3 and 4 amplify fragments of 832 and 647 bp, respectively, spanning genomic sequence of the wild-type allele that includes the 5′ and 3′ arms used for homologous recombination.

For Southern blot genotyping of ES cells and mice, genomic DNA was isolated by phenol/chloroform and digested as described. Probes for Southern hybridization were generated by PCR amplification using the following primers: 5′ flanking probe I, Fw TTAGGGGAGGGAAGGCTTTA, Rv CGGCATCGCTTATTGAAGAT, 60°C annealing, 311 bp; 3′ flanking probe II, AAGATAAGGACATGTTAGCC, Rv TCTGTGGAGCTTAATATCTG, 60°C annealing, 313 bp; *Hprt* internal probe III, Fw CTGGGTCAAGGGGAAAGAGT, Rv GAGGTGAGGTGGGAAAATCA, 60°C annealing, 704 bp. Probe I detects fragments of 13,331 and 15,875 bp from the wild-type and mutant allele, respectively, upon digestion with SwaI and EcoRI; probe II identifies fragments of 8441 and 11,648 bp from the wild-type and mutant allele, respectively, following digestion with XhoI and AflII; probe III reveals a fragment of 8653 bp from the mutant allele only, upon digestion with I-PpoI (Fig. 2).

### Tissue collection, RNA extraction, and cDNA synthesis

Tissues from embryos and adult animals were snap-frozen in liquid nitrogen following quick dissection after uterine collection or humane sacrifice, respectively. Total RNA was isolated with TRIzol (Invitrogen) according to the manufacturer's instructions, quantified by spectrophotometry, and quality controlled by gel electrophoresis to assess the integrity of the ribosomal RNA and absence of degradation products.

Two micrograms of total RNA were treated with 1 unit of RQ1 DNase (Promega) to remove genomic DNA contaminants and were reverse transcribed using the RevertAid H Minus cDNA synthesis kit (Fermentas) with random primers. The resulting cDNA was diluted 1:20, aliquoted, and stored at −20°C for subsequent use.

To discriminate between *Rtl1* and *Rtl1* anti-sense (*Rtl1-AS*) transcripts, a strategy was adopted whereby M13-R and Mp-F “adaptor” sequences, which are not found in the mouse genome, were tethered to *Rtl1-AS*-specific primers. Strand-specific reverse transcription was then performed as described above with adaptor-containing primers 5′-TGTCAGGCAACCGTATTCACC**GGAGTCCAGCGATGGTTCAC**-3′, where the nucleotides in bold are the gene-specific sequence.

For the synthesis of miR-127 and miR-433 cDNA, reverse transcription was carried out in 6-µL reactions made up with 1 µg total RNA, 1× RT reaction buffer (Fermentas), 1 mM dNTPs (each), 1× RT microRNA primer (ABI), 100 units RevertAid H-minus reverse transcriptase (Fermentas), and 20 units RiboLock RNase inhibitor (Fermentas). The microRNA primers were used in multiplex in the RT reaction, which included the control small RNA snoRNA 202. The reaction mixture was covered with 10 µL mineral oil to prevent evaporation, incubated for 30 min at 16°C, for 30 min at 42°C, and for 5 min at 85°C to inactivate the enzymes. The cDNA was extracted following the addition of 44 µL distilled water and 20 µL chloroform, vigorous vortexing, and centrifugation at 1000 rcf.

### Analysis of allelic expression

Wild-type and heterozygous embryos with expressed polymorphisms at Chromosome 12 were obtained from reciprocal crosses between *del*^L1rep^ heterozygous mutants (C57BL/6 background) and *Mus musculus castaneus* (strain CAST/EiJ, abbreviated Cast) (Jackson laboratories) or *Mus musculus molossinus* 12 (strain B6.MOLF12A, abbreviated Mol 12) (Supplemental Table S7). The B6.MOLF12A congenic strain carries an 8 cM region of Chromosome 12 from the *Mus musculus molossinus* strain MOLF/Ei on a C57BL/6 genetic background. Allelic expression of eight transcripts was assessed by PCR amplification of fetal (E16.5) brain, liver, and placenta cDNA with biotinylated primers, followed by pyrosequencing. Primer sequences for PCR amplification of the analyzed polymorphisms and amplification conditions are shown in Supplemental Table S7. Pyrosequencing was carried out with up to 15 µL of PCR product. The biotinylated strand was purified using Strepdavidin Sepharose High Performance Beads (GE Healthcare), washed, denatured, and annealed to the pyrosequencing primer (0.3 µM) using PyroMark reagents (Qiagen), as described (Tost and Gut 2007). Pyrosequencing was performed on a PyroMark MD pyrosequencer (Biotage) using PyroMark Gold Qp6 SQA reagents (Roche), and the data were analyzed with the PyroMark MD version 1.0 software (Biotage).

### Gene expression studies

Real-time quantitative PCR with SYBR Green was performed to measure the levels of gene expression in fetal brain, liver, and placenta, and in adult brain. Target gene expression was normalized to the expression of *Gap3dh* for brain, and of *beta-2-microglobulin* for liver and placenta. Thermocycling was performed on a DNA engine Opticon 2 thermocycler (MJ Research) in 12.5-µL reactions with SensiMix (Quantace), 400 nM of primers (except where noted) and 3 µL of cDNA synthesized and diluted as described above, in duplicate. A standard curve made up of doubling dilutions of pooled cDNA from the samples being assayed was run on each plate, and quantification was performed relative to the standard curve. All PCR products were subsequently checked for specificity by gel electrophoresis. Primer sequences and annealing temperatures are shown in Supplemental Table S8. All primers amplified with an estimated efficiency of 80%–110%.

The expression levels of miR-127 and miR-433 were measured by TaqMan assays. Ten-microliter reactions were made up according to manufacturer's instructions but scaled down: 1× TaqMan Universal PCR mastermix (ABI), 1× microRNAqPCR primer (ABI), and 2 µL diluted cDNA, synthesized as described. Thermocycling was performed on an Applied Biosystems 7500 Fast Real-Time PCR system with 10 min at 95°C and 40 cycles for 15 sec at 95°C and for 60 sec at 60°C. Data were normalized to snoRNA202 expression and quantified using the ΔΔCt method.

### DLK1 Protein quantification by ELISA

Adult brains from wild-type, heterozygous, and homozygous *del*^L1rep^ animals were homogenized in 1 mL RIPA buffer, and 25 µL were assayed as previously described (Charalambous et al. 2012).

### Methylation analyses

Sodium bisulphite mutagenesis was carried out on 1 µg gDNA per sample using the two-step conversion protocol of the Imprint DNA Modification Kit (Sigma). Two samples with no template were included to confirm contamination had not occurred during bisulphite treatment. Five microliters of bisulphite-treated sample were PCR amplified with biotinylated primers (Forward 5′-GTGGTTTGTTATGGGTAAGTTT, Reverse 5′-Btn-CCCTTCCCTCACTCCAAAAATTAA, 54°C annealing for 36 cycles) and quality controlled by gel electrophoresis of 5 µL of each PCR reaction. A volume of up to 15 µL PCR product was then used for pyrosequencing, which was carried out as previously described (Tost and Gut 2007). The biotinylated strand was purified using Strepdavidin Sepharose High Performance Beads (GE Healthcare), washed, denatured, and annealed to the pyrosequencing primer (0.3 µM 5′-TGGTTTATTGTATATAATGT) using PyroMark reagents (Qiagen). Pyrosequencing was performed on a PyroMark MD pyrosequencer (Biotage) using PyroMark Gold Qp6 SQA reagents (Roche), and the data were analyzed with the Pyro Q-CpG 1.0.9 software (Biotage).

### LINE-1 transcription studies

Primers for amplification of LINE-1 elements specific to the deleted interval on Chromosome 12 were designed by taking the alignment between the L1 of interest and the L1 consensus sequence from RepeatMasker (Smit et al. 2015) and identifying regions of low conservation. Primer pairs were designed to the low conservation regions using Primer3 (Koressaar and Remm 2007; Untergasser et al. 2012) then tested for successful amplification and specificity using genomic DNA of wild-type and *del*^L1rep^ homozygous mice, respectively. Primer sequences and PCR conditions are as shown in Supplemental Table S9.

Transcription was examined by real-time PCR amplification of 3 µL cDNA from fetal brain and liver, and adult brain (prepared and diluted as described above), in a 12.5-µL reaction containing SensiMix (Quantace) and 200 nM primer. Reactions were carried out on a DNA engine Opticon 2 thermocycler (MJ Research) and subsequently checked by gel by electrophoresis. To assess the allele-specific mode of transcription, cDNA from fetal brain from embryos with uniparental duplications of Chromosome 12 (matUPD12 and patUPD12) (Georgiades et al. 2000) was used and amplified as above.

### In vitro luciferase assays

DNA fragments were generated by PCR from mouse gDNA using primers designed with either two SacI or two NheI restriction sites at each end in order to clone both in sense and antisense orientation. Each 25-µL reaction contained 1× PCR Buffer (KOD Hot Start, Novagen), 300 µM dNTPs, 1 mM MgSO_4_, 0.5 units Hot Start KOD polymerase, 0.6 µM of each primer, and 50 ng of template. The cycling parameters were as follows: for 2 min at 94°C, 35 cycles for 15 seconds at 94°C, annealing temperature (specific for each primer) for 30 sec, 72°C for 1 min/kb of expected product, and a 5 min extension cycle at 72°C. PCR products were digested with appropriate restriction enzyme and cloned into pGL3 Promoter (Promega) in both orientations using standard molecular techniques. PCR primers and conditions are as shown in Supplemental Table S10.

Transfections were performed by electroporation (1500 V, 20 msec, 1 pulse), and 0.3 µg luciferase construct was cotransfected with 0.1 µg pSV40PK *Renilla* (Promega) into 2 × 10^5^ neurospheres derived from E16.5 brains as described (Ferrón et al. 2007). Neurospheres were cultured in RHB basal media (Takara) supplemented with N2, Gentamycin, BSA, EGF, and FGF. A positive pGL3 Control vector (Promega) containing both the SV40 promoter and enhancer was transfected to test the assay conditions, while the pSV40PK *Renilla* vector alone demonstrated no firefly luciferase activity and acted as an internal control. Each construct was tested in triplicate per plate.

For mouse embryonic fibroblast cell line (NIH-3T3) and mouse embryonic stem cell line (E14Tg2a), cells were cultured under standard/normal tissue culture conditions. On the day prior to transfection, cells were plated into 48-well plates at a concentration of 1–2 × 10^4^ cells per well, and 0.5 µg plasmid DNA was added to serum free media with 1 unit of transfection reagent TurboFect (Fermentas) in a 50-µL total reaction. The reaction was incubated at room temperature for 15–20 min then added dropwise to the media in the well. The media was changed 6 h after transfection, and the reaction was left for 48 h (for ES cells, media was changed daily). For neural stem cells derived from a mouse embryonic stem cell line, 46C (Ying et al. 2003) cells were cultured in RHB A complete media (StemCells) supplemented with EGF and FGF. 1.5–3 × 10^4^ cells per well were seeded into 48 well plates. After 24 h, 0.5 µg DNA was added to 25 µL diluent and 1.6 µL Nanofectin (PAA) to 25 µL diluent, then combined. This reaction was then added dropwise to the media in each well. The media was changed 3–4 h after transfection.

After 48 h, cells were tested for their luciferase activity using the Dual-Luciferase Reporter (DLR) Assay System (Promega) according to the manufacturer's protocol. Each cell lysate was measured in duplicate on a single channel TD20/20 Luminometer (Turner Designs). The luminometer was programmed with a 2-sec pre-read delay, followed by a 10-sec measurement period. The firefly luciferase values were normalized to the *Renilla* values, and then each test construct was normalized to pGL3-Promoter. Statistics for the in vitro luciferase reporter assays were calculated by the one-way ANOVA (and nonparametric) Friedman test to calculate the overall statistical significance (*P*-value), followed by Dunn's Multiple Comparison post-test to the empty vector to calculate the statistical significance (*P*-value) between specific samples. *P* < 0.05 was deemed as statistically significant.

### Statistical analyses

All statistical tests were performed using the GraphPad Prism Software Version 5.00 for Windows (GraphPad Software; https://www.graphpad.com) or R (R Core Team 2015) and are indicated in the respective section of the text or in figure legends. Figures show the mean, and error bars represent the standard error of the mean. Significance values and the number of samples analyzed (*n*) are indicated in the respective figure legends. *P* < 0.05 was deemed as statistically significant.

## Supplementary Material

Supplemental Material

## References

[GR221366SOAC1] Adams DJ, Biggs PJ, Cox T, Davies R, van der Weyden L, Jonkers J, Smith J, Plumb B, Taylor R, Nishijima I, 2004 Mutagenic insertion and chromosome engineering resource (MICER). Nat Genet 36: 867–871. 15235602 10.1038/ng1388

[GR221366SOAC2] Allen E, Horvath S, Tong F, Kraft P, Spiteri E, Riggs AD, Marahrens Y. 2003 High concentrations of long interspersed nuclear element sequence distinguish monoallelically expressed genes. Proc Natl Acad Sci 100: 9940–9945. 12909712 10.1073/pnas.1737401100 PMC187893

[GR221366SOAC3] Baillie JK, Barnett MW, Upton KR, Gerhardt DJ, Richmond TA, De Sapio F, Brennan PM, Rizzu P, Smith S, Fell M, 2011 Somatic retrotransposition alters the genetic landscape of the human brain. Nature 479: 534–537. 22037309 10.1038/nature10531 PMC3224101

[GR221366SOAC4] Beck CR, Collier P, Macfarlane C, Malig M, Kidd JM, Eichler EE, Badge RM, Moran JV. 2010 LINE-1 retrotransposition activity in human genomes. Cell 141: 1159–1170. 20602998 10.1016/j.cell.2010.05.021 PMC3013285

[GR221366SOAC5] Belancio VP, Hedges DJ, Deininger P. 2006 LINE-1 RNA splicing and influences on mammalian gene expression. Nucleic Acids Res 34: 1512–1521. 16554555 10.1093/nar/gkl027 PMC1415225

[GR221366SOAC6] Blanchette M, Kent WJ, Riemer C, Elnitski L, Smit AF, Roskin KM, Baertsch R, Rosenbloom K, Clawson H, Green ED, 2004 Aligning multiple genomic sequences with the threaded blockset aligner. Genome Res 14: 708–715. 15060014 10.1101/gr.1933104 PMC383317

[GR221366SOAC7] Blankenberg D, Von Kuster G, Coraor N, Ananda G, Lazarus R, Mangan M, Nekrutenko A, Taylor J. 2010 Galaxy: a web-based genome analysis tool for experimentalists. Curr Protoc Mol Biol **Chapter 19:** Unit 19.10.1-21. 10.1002/0471142727.mb1910s89 PMC4264107 20069535

[GR221366SOAC8] Bogerd HP, Wiegand HL, Hulme AE, Garcia-Perez JL, O'Shea KS, Moran JV, Cullen BR. 2006 Cellular inhibitors of long interspersed element 1 and Alu retrotransposition. Proc Natl Acad Sci 103: 8780–8785. 16728505 10.1073/pnas.0603313103 PMC1482655

[GR221366SOAC9] Bourc'his D, Bestor TH. 2004 Meiotic catastrophe and retrotransposon reactivation in male germ cells lacking Dnmt3L. Nature 431: 96–99. 15318244 10.1038/nature02886

[GR221366SOAC10] Branciforte D, Martin SL. 1994 Developmental and cell type specificity of LINE-1 expression in mouse testis: implications for transposition. Mol Cell Biol 14: 2584–2592. 8139560 10.1128/mcb.14.4.2584-2592.1994 PMC358626

[GR221366SOAC11] Brouha B, Schustak J, Badge RM, Lutz-Prigge S, Farley AH, Moran JV, Kazazian HH Jr. 2003 Hot L1s account for the bulk of retrotransposition in the human population. Proc Natl Acad Sci 100: 5280–5285. 12682288 10.1073/pnas.0831042100 PMC154336

[GR221366SOAC12] Charalambous M, Ferrón SR, da Rocha ST, Murray AJ, Rowland T, Ito M, Schuster-Gossler K, Hernandez A, Ferguson-Smith AC. 2012 Imprinted gene dosage is critical for the transition to independent life. Cell Metab 15: 209–221. 22326222 10.1016/j.cmet.2012.01.006 PMC3314949

[GR221366SOAC13] Chen H, Lilley CE, Yu Q, Lee DV, Chou J, Narvaiza I, Landau NR, Weitzman MD. 2006 APOBEC3A is a potent inhibitor of adeno-associated virus and retrotransposons. Curr Biol 16: 480–485. 16527742 10.1016/j.cub.2006.01.031

[GR221366SOAC14] Chow JC, Ciaudo C, Fazzari MJ, Mise N, Servant N, Glass JL, Attreed M, Avner P, Wutz A, Barillot E, 2010 LINE-1 activity in facultative heterochromatin formation during X chromosome inactivation. Cell 141: 956–969. 20550932 10.1016/j.cell.2010.04.042

[GR221366SOAC15] Cordaux R, Batzer MA. 2009 The impact of retrotransposons on human genome evolution. Nat Rev Genet 10: 691–703. 19763152 10.1038/nrg2640 PMC2884099

[GR221366SOAC16] Coufal NG, Garcia-Perez JL, Peng GE, Yeo GW, Mu Y, Lovci MT, Morell M, O'Shea KS, Moran JV, Gage FH. 2009 L1 retrotransposition in human neural progenitor cells. Nature 460: 1127–1131. 19657334 10.1038/nature08248 PMC2909034

[GR221366SOAC17] DeBerardinis RJ, Goodier JL, Ostertag EM, Kazazian HH Jr. 1998 Rapid amplification of a retrotransposon subfamily is evolving the mouse genome. Nat Genet 20: 288–290. 9806550 10.1038/3104

[GR221366SOAC18] The ENCODE Project Consortium. 2011 A user's guide to the encyclopedia of DNA elements (ENCODE). PLoS Biol 9: e1001046. 21526222 10.1371/journal.pbio.1001046 PMC3079585

[GR221366SOAC19] Evrony GD, Cai X, Lee E, Hills LB, Elhosary PC, Lehmann HS, Parker JJ, Atabay KD, Gilmore EC, Poduri A, 2012 Single-neuron sequencing analysis of L1 retrotransposition and somatic mutation in the human brain. Cell 151: 483–496. 23101622 10.1016/j.cell.2012.09.035 PMC3567441

[GR221366SOAC20] Ferguson-Smith AC. 2011 Genomic imprinting: the emergence of an epigenetic paradigm. Nat Rev Genet 12: 565–575. 21765458 10.1038/nrg3032

[GR221366SOAC21] Ferrón SR, Andreu-Agulló C, Mira H, Sánchez P, Marqués-Torrejón MA, Fariñas I. 2007 A combined *ex*/*in vivo* assay to detect effects of exogenously added factors in neural stem cells. Nat Protoc 2: 849–859. 17474182 10.1038/nprot.2007.104

[GR221366SOAC22] Ferrón SR, Charalambous M, Radford E, McEwen K, Wildner H, Hind E, Morante-Redolat JM, Laborda J, Guillemot F, Bauer SR, 2011 Postnatal loss of *Dlk1* imprinting in stem cells and niche astrocytes regulates neurogenesis. Nature 475: 381–385. 21776083 10.1038/nature10229 PMC3160481

[GR221366SOAC23] Finlan LE, Sproul D, Thomson I, Boyle S, Kerr E, Perry P, Ylstra B, Chubb JR, Bickmore WA. 2008 Recruitment to the nuclear periphery can alter expression of genes in human cells. PLoS Genet 4: e1000039. 18369458 10.1371/journal.pgen.1000039 PMC2265557

[GR221366SOAC24] Georgiades P, Watkins M, Surani MA, Ferguson-Smith AC. 2000 Parental origin-specific developmental defects in mice with uniparental disomy for chromosome 12. Development 127: 4719–4728. 11023874 10.1242/dev.127.21.4719

[GR221366SOAC25] Giardine B, Riemer C, Hardison RC, Burhans R, Elnitski L, Shah P, Zhang Y, Blankenberg D, Albert I, Taylor J, 2005 Galaxy: a platform for interactive large-scale genome analysis. Genome Res 15: 1451–1455. 16169926 10.1101/gr.4086505 PMC1240089

[GR221366SOAC26] Gilbert N, Lutz-Prigge S, Moran JV. 2002 Genomic deletions created upon LINE-1 retrotransposition. Cell 110: 315–325. 12176319 10.1016/s0092-8674(02)00828-0

[GR221366SOAC27] Gilbert N, Lutz S, Morrish TA, Moran JV. 2005 Multiple fates of L1 retrotransposition intermediates in cultured human cells. Mol Cell Biol 25: 7780–7795. 16107723 10.1128/MCB.25.17.7780-7795.2005 PMC1190285

[GR221366SOAC28] Girard A, Sachidanandam R, Hannon GJ, Carmell MA. 2006 A germline-specific class of small RNAs binds mammalian Piwi proteins. Nature 442: 199–202. 16751776 10.1038/nature04917

[GR221366SOAC29] Goecks J, Nekrutenko A, Taylor J. 2010 Galaxy: a comprehensive approach for supporting accessible, reproducible, and transparent computational research in the life sciences. Genome Biol 11: R86. 20738864 10.1186/gb-2010-11-8-r86 PMC2945788

[GR221366SOAC30] Goodier JL, Ostertag EM, Du K, Kazazian HH Jr. 2001 A novel active L1 retrotransposon subfamily in the mouse. Genome Res 11: 1677–1685. 11591644 10.1101/gr.198301 PMC311137

[GR221366SOAC31] Grant J, Mahadevaiah SK, Khil P, Sangrithi MN, Royo H, Duckworth J, McCarrey JR, Vandeberg JL, Renfree MB, Taylor W, 2012 *Rsx* is a metatherian RNA with *Xist*-like properties in X-chromosome inactivation. Nature 487: 254–258. 22722828 10.1038/nature11171 PMC3484893

[GR221366SOAC32] Hagan JP, O'Neill BL, Stewart CL, Kozlov SV, Croce CM. 2009 At least ten genes define the imprinted *Dlk1-Dio3* cluster on mouse chromosome 12qF1. PLoS One 4: e4352. 19194500 10.1371/journal.pone.0004352 PMC2632752

[GR221366SOAC33] Hambor JE, Mennone J, Coon ME, Hanke JH, Kavathas P. 1993 Identification and characterization of an *Alu*-containing, T-cell-specific enhancer located in the last intron of the human CD8α gene. Mol Cell Biol 13: 7056–7070. 8413295 10.1128/mcb.13.11.7056-7070.1993 PMC364767

[GR221366SOAC34] Han JS, Szak ST, Boeke JD. 2004 Transcriptional disruption by the L1 retrotransposon and implications for mammalian transcriptomes. Nature 429: 268–274. 15152245 10.1038/nature02536

[GR221366SOAC35] Han K, Lee J, Meyer TJ, Remedios P, Goodwin L, Batzer MA. 2008 L1 recombination-associated deletions generate human genomic variation. Proc Natl Acad Sci 105: 19366–19371. 19036926 10.1073/pnas.0807866105 PMC2614767

[GR221366SOAC36] Huang CR, Schneider AM, Lu Y, Niranjan T, Shen P, Robinson MA, Steranka JP, Valle D, Civin CI, Wang T, 2010 Mobile interspersed repeats are major structural variants in the human genome. Cell 141: 1171–1182. 20602999 10.1016/j.cell.2010.05.026 PMC2943426

[GR221366SOAC37] Hutter B, Bieg M, Helms V, Paulsen M. 2010 Imprinted genes show unique patterns of sequence conservation. BMC Genomics 11: 649. 21092170 10.1186/1471-2164-11-649 PMC3091771

[GR221366SOAC38] International Human Genome Sequencing Consortium. 2001 Initial sequencing and analysis of the human genome. Nature 409: 860–921. 11237011 10.1038/35057062

[GR221366SOAC39] Iskow RC, McCabe MT, Mills RE, Torene S, Pittard WS, Neuwald AF, Van Meir EG, Vertino PM, Devine SE. 2010 Natural mutagenesis of human genomes by endogenous retrotransposons. Cell 141: 1253–1261. 20603005 10.1016/j.cell.2010.05.020 PMC2943760

[GR221366SOAC40] Kano H, Godoy I, Courtney C, Vetter MR, Gerton GL, Ostertag EM, Kazazian HH Jr. 2009 L1 retrotransposition occurs mainly in embryogenesis and creates somatic mosaicism. Genes Dev 23: 1303–1312. 19487571 10.1101/gad.1803909 PMC2701581

[GR221366SOAC41] Karolchik D, Hinrichs AS, Furey TS, Roskin KM, Sugnet CW, Haussler D, Kent WJ. 2004 The UCSC Table Browser data retrieval tool. Nucleic Acids Res 32: D493–D496. 14681465 10.1093/nar/gkh103 PMC308837

[GR221366SOAC42] Kent WJ, Sugnet CW, Furey TS, Roskin KM, Pringle TH, Zahler AM, Haussler D. 2002 The human genome browser at UCSC. Genome Res 12: 996–1006. 12045153 10.1101/gr.229102 PMC186604

[GR221366SOAC43] Koressaar T, Remm M. 2007 Enhancements and modifications of primer design program Primer3. Bioinformatics 23: 1289–1291. 17379693 10.1093/bioinformatics/btm091

[GR221366SOAC44] Kota SK, Llères D, Bouschet T, Hirasawa R, Marchand A, Begon-Pescia C, Sanli I, Arnaud P, Journot L, Girardot M, 2014 ICR noncoding RNA expression controls imprinting and DNA replication at the *Dlk1-Dio3* domain. Dev Cell 31: 19–33. 25263792 10.1016/j.devcel.2014.08.009

[GR221366SOAC45] Kuramochi-Miyagawa S, Watanabe T, Gotoh K, Totoki Y, Toyoda A, Ikawa M, Asada N, Kojima K, Yamaguchi Y, Ijiri TW, 2008 DNA methylation of retrotransposon genes is regulated by Piwi family members MILI and MIWI2 in murine fetal testes. Genes Dev 22: 908–917. 18381894 10.1101/gad.1640708 PMC2279202

[GR221366SOAC46] Lin SP, Youngson N, Takada S, Seitz H, Reik W, Paulsen M, Cavaille J, Ferguson-Smith AC. 2003 Asymmetric regulation of imprinting on the maternal and paternal chromosomes at the *Dlk1-Gtl2* imprinted cluster on mouse chromosome 12. Nat Genet 35: 97–102. 12937418 10.1038/ng1233

[GR221366SOAC47] Long Q, Bengra C, Li C, Kutlar F, Tuan D. 1998 A long terminal repeat of the human endogenous retrovirus ERV-9 is located in the 5′ boundary area of the human β-globin locus control region. Genomics 54: 542–555. 9878258 10.1006/geno.1998.5608

[GR221366SOAC48] Lyon MF. 1998 X-chromosome inactivation: a repeat hypothesis. Cytogenet Cell Genet 80: 133–137. 9678347 10.1159/000014969

[GR221366SOAC49] Malik HS, Burke WD, Eickbush TH. 1999 The age and evolution of non-LTR retrotransposable elements. Mol Biol Evol 16: 793–805. 10368957 10.1093/oxfordjournals.molbev.a026164

[GR221366SOAC50] Meuleman W, Peric-Hupkes D, Kind J, Beaudry JB, Pagie L, Kellis M, Reinders M, Wessels L, van Steensel B. 2013 Constitutive nuclear lamina–genome interactions are highly conserved and associated with A/T-rich sequence. Genome Res 23: 270–280. 23124521 10.1101/gr.141028.112 PMC3561868

[GR221366SOAC51] Mikkelsen TS, Wakefield MJ, Aken B, Amemiya CT, Chang JL, Duke S, Garber M, Gentles AJ, Goodstadt L, Heger A, 2007 Genome of the marsupial *Monodelphis domestica* reveals innovation in non-coding sequences. Nature 447: 167–177. 17495919 10.1038/nature05805

[GR221366SOAC52] Mouse Genome Sequencing Consortium. 2002 Initial sequencing and comparative analysis of the mouse genome. Nature 420: 520–562. 12466850 10.1038/nature01262

[GR221366SOAC53] Muckenfuss H, Hamdorf M, Held U, Perković M, Löwer J, Cichutek K, Flory E, Schumann GG, Münk C. 2006 APOBEC3 proteins inhibit human LINE-1 retrotransposition. J Biol Chem 281: 22161–22172. 16735504 10.1074/jbc.M601716200

[GR221366SOAC54] Muotri AR, Chu VT, Marchetto MC, Deng W, Moran JV, Gage FH. 2005 Somatic mosaicism in neuronal precursor cells mediated by L1 retrotransposition. Nature 435: 903–910. 15959507 10.1038/nature03663

[GR221366SOAC55] Muotri AR, Zhao C, Marchetto MC, Gage FH. 2009 Environmental influence on L1 retrotransposons in the adult hippocampus. Hippocampus 19: 1002–1007. 19771587 10.1002/hipo.20564 PMC2758700

[GR221366SOAC56] O'Gorman S, Fox DT, Wahl GM. 1991 Recombinase-mediated gene activation and site-specific integration in mammalian cells. Science 251: 1351–1355. 1900642 10.1126/science.1900642

[GR221366SOAC57] Ostertag EM, Kazazian HH Jr. 2001 Biology of mammalian L1 retrotransposons. Annu Rev Genet 35: 501–538. 11700292 10.1146/annurev.genet.35.102401.091032

[GR221366SOAC58] Penzkofer T, Dandekar T, Zemojtel T. 2005 L1Base: from functional annotation to prediction of active LINE-1 elements. Nucleic Acids Res 33: D498–D500. 15608246 10.1093/nar/gki044 PMC539998

[GR221366SOAC59] Penzkofer T, Jäger M, Figlerowicz M, Badge R, Mundlos S, Robinson PN, Zemojtel T. 2016 L1Base 2: more retrotransposition-active LINE-1s, more mammalian genomes. Nucleic Acids Res 45: D68–D73. 27924012 10.1093/nar/gkw925 PMC5210629

[GR221366SOAC60] Peric-Hupkes D, Meuleman W, Pagie L, Bruggeman SW, Solovei I, Brugman W, Gräf S, Flicek P, Kerkhoven RM, van Lohuizen M, 2010 Molecular maps of the reorganization of genome-nuclear lamina interactions during differentiation. Mol Cell 38: 603–613. 20513434 10.1016/j.molcel.2010.03.016 PMC5975946

[GR221366SOAC61] Quinlan AR, Hall IM. 2010 BEDTools: a flexible suite of utilities for comparing genomic features. Bioinformatics 26: 841–842. 20110278 10.1093/bioinformatics/btq033 PMC2832824

[GR221366SOAC018] R Core Team. 2015 R: a language and environment for statistical computing. R Foundation for Statistical Computing, Vienna, Austria. https://www.R-project.org/.

[GR221366SOAC62] Richardson SR, Narvaiza I, Planegger RA, Weitzman MD, Moran JV. 2014 APOBEC3A deaminates transiently exposed single-strand DNA during LINE-1 retrotransposition. eLife 3: e02008. 24843014 10.7554/eLife.02008 PMC4003774

[GR221366SOAC63] Shirohzu H, Yokomine T, Sato C, Kato R, Toyoda A, Purbowasito W, Suda C, Mukai T, Hattori M, Okumura K, 2004 A 210-kb segment of tandem repeats and retroelements located between imprinted subdomains of mouse distal chromosome 7. DNA Res 11: 325–334. 15747580 10.1093/dnares/11.5.325

[GR221366SOAC64] Simons C, Pheasant M, Makunin IV, Mattick JS. 2006 Transposon-free regions in mammalian genomes. Genome Res 16: 164–172. 16365385 10.1101/gr.4624306 PMC1361711

[GR221366SOAC65] Smit MA, Tordoir X, Gyapay G, Cockett NE, Georges M, Charlier C. 2005 *BEGAIN*: a novel imprinted gene that generates paternally expressed transcripts in a tissue- and promoter-specific manner in sheep. Mamm Genome 16: 801–814. 16261422 10.1007/s00335-004-2415-z

[GR221366SOAC66] Smit AFA, Hubley R, Green P. 2015 RepeatMasker Open-4.0. http://www.repeatmasker.org.

[GR221366SOAC67] Song G, Wang L. 2008 MiR-433 and miR-127 arise from independent overlapping primary transcripts encoded by the miR-433-127 locus. PLoS One 3: e3574. 18974780 10.1371/journal.pone.0003574 PMC2570487

[GR221366SOAC68] Speek M. 2001 Antisense promoter of human L1 retrotransposon drives transcription of adjacent cellular genes. Mol Cell Biol 21: 1973–1985. 11238933 10.1128/MCB.21.6.1973-1985.2001 PMC86790

[GR221366SOAC69] Symer DE, Connelly C, Szak ST, Caputo EM, Cost GJ, Parmigiani G, Boeke JD. 2002 Human l1 retrotransposition is associated with genetic instability in vivo. Cell 110: 327–338. 12176320 10.1016/s0092-8674(02)00839-5

[GR221366SOAC70] Tost J, Gut IG. 2007 DNA methylation analysis by pyrosequencing. Nat Protoc 2: 2265–2275. 17853883 10.1038/nprot.2007.314

[GR221366SOAC71] Untergasser A, Cutcutache I, Koressaar T, Ye J, Faircloth BC, Remm M, Rozen SG. 2012 Primer3—new capabilities and interfaces. Nucleic Acids Res 40: e115. 22730293 10.1093/nar/gks596 PMC3424584

[GR221366SOAC72] Upton KR, Gerhardt DJ, Jesuadian JS, Richardson SR, Sánchez-Luque FJ, Bodea GO, Ewing AD, Salvador-Palomeque C, van der Knaap MS, Brennan PM, 2015 Ubiquitous L1 mosaicism in hippocampal neurons. Cell 161: 228–239. 25860606 10.1016/j.cell.2015.03.026 PMC4398972

[GR221366SOAC73] Verley FA, Grahn D, Leslie WP, Hamilton KF. 1967 Sex ratio of mice as possible indicator of mutation rate for sex-linked lethals. J Hered 58: 285–290. 5590152 10.1093/oxfordjournals.jhered.a107616

[GR221366SOAC74] Visel A, Blow MJ, Li Z, Zhang T, Akiyama JA, Holt A, Plajzer-Frick I, Shoukry M, Wright C, Chen F, 2009 ChIP-seq accurately predicts tissue-specific activity of enhancers. Nature 457: 854–858. 19212405 10.1038/nature07730 PMC2745234

[GR221366SOAC75] Wade DP, Puckey LH, Knight BL, Acquati F, Mihalich A, Taramelli R. 1997 Characterization of multiple enhancer regions upstream of the apolipoprotein(a) gene. J Biol Chem 272: 30387–30399. 9374529 10.1074/jbc.272.48.30387

[GR221366SOAC76] Watanabe T, Takeda A, Tsukiyama T, Mise K, Okuno T, Sasaki H, Minami N, Imai H. 2006 Identification and characterization of two novel classes of small RNAs in the mouse germline: retrotransposon-derived siRNAs in oocytes and germline small RNAs in testes. Genes Dev 20: 1732–1743. 16766679 10.1101/gad.1425706 PMC1522070

[GR221366SOAC77] Yang N, Kazazian HH Jr. 2006 L1 retrotransposition is suppressed by endogenously encoded small interfering RNAs in human cultured cells. Nat Struct Mol Biol 13: 763–771. 16936727 10.1038/nsmb1141

[GR221366SOAC78] Yang Z, Boffelli D, Boonmark N, Schwartz K, Lawn R. 1998 Apolipoprotein(a) gene enhancer resides within a LINE element. J Biol Chem 273: 891–897. 9422746 10.1074/jbc.273.2.891

[GR221366SOAC79] Ying QL, Stavridis M, Griffiths D, Li M, Smith A. 2003 Conversion of embryonic stem cells into neuroectodermal precursors in adherent monoculture. Nat Biotechnol 21: 183–186. 12524553 10.1038/nbt780

[GR221366SOAC80] Yoder JA, Walsh CP, Bestor TH. 1997 Cytosine methylation and the ecology of intragenomic parasites. Trends Genet 13: 335–340. 9260521 10.1016/s0168-9525(97)01181-5

[GR221366SOAC81] Younger RM, Amadou C, Bethel G, Ehlers A, Lindahl KF, Forbes S, Horton R, Milne S, Mungall AJ, Trowsdale J, 2001 Characterization of clustered MHC-linked olfactory receptor genes in human and mouse. Genome Res 11: 519–530. 11282967 10.1101/gr.160301 PMC311051

