# Supplementary material for: Targeted deletion of a 170-kb cluster of LINE-1 repeats and implications for regional control
Source: Genome Res. 2018 Mar;28(3):345–56. doi: 10.1101/gr.221366.117 (PMC5848613; doi:10.1101/gr.221366.117)
Supplement: Supplemental Material [file supp_28_3_345__index.html]

Targeted deletion of a 170-kb cluster of LINE-1 repeats and implications for regional control — Supplemental Material 

# Targeted deletion of a 170-kb cluster of LINE-1 repeats and implications for regional control

## Supplemental Material

- Supplemental\_Material.pdf
