# Supplementary material for: Targeted deletion of a 170-kb cluster of LINE-1 repeats and implications for regional control
Source: Genome Res. 2018 Mar;28(3):345–56. doi: 10.1101/gr.221366.117 (PMC5848613; doi:10.1101/gr.221366.117)
Supplement: Supplemental Material [file supp_gr.221366.117_Supplemental_Material.pdf]

Supplemental Material

Table of Contents

Supplemental Fig S1	2
Supplemental Fig S2	4
Supplemental Fig S3	5
Supplemental Fig S4	6
Supplemental Fig S5	7
Supplemental Fig S6	9
Supplemental Fig S7	11
Supplemental Fig S8	13
Supplemental Fig S9	15
Supplemental Fig S10	17
Supplemental Fig S11	19
Supplemental Table S1	20
Supplemental Table S2	22
Supplemental Table S3	24
Supplemental Table S4	26
Supplemental Table S5	27
Supplemental Table S6	28
Supplemental Table S7	29
Supplemental Table S8	30
Supplemental Table S9	31
Supplemental Table S10	32
Supplemental References	33

Supplemental Fig S1

A

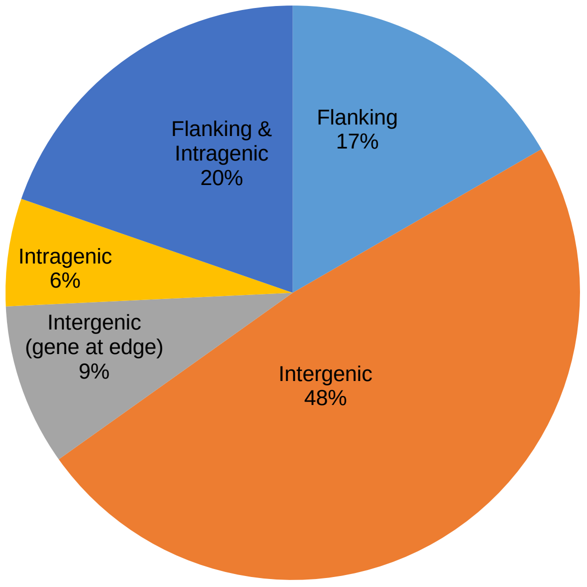

B

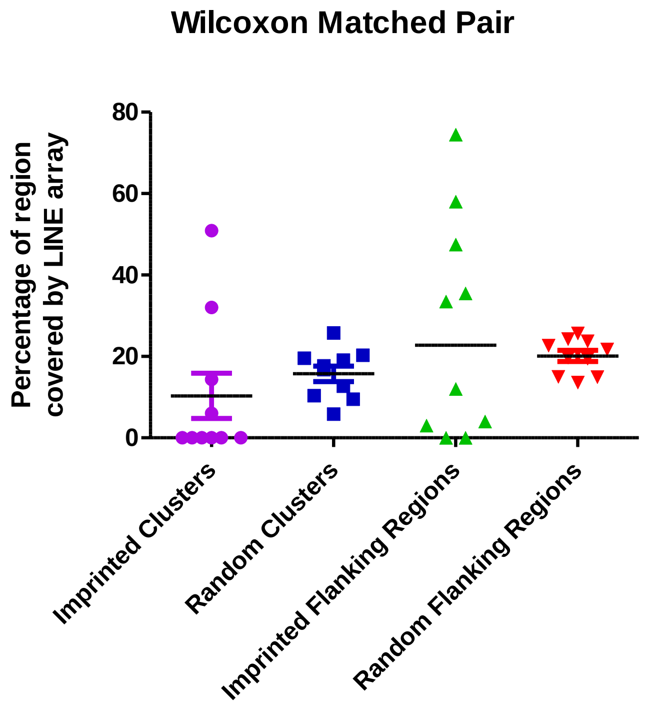

Supplemental Fig S1. (A) **Genomic context of the LINE dense arrays of >70 % LINEs covering >100 Kb within the mouse genome**. Intragenic = LINE array falls within a gene, Flanking = LINE array surrounds small genes in the region, Flanking & Intragenic = LINE array surrounds and is located within genes in the region, Intergenic = LINE array falls between genes, Intergenic (gene at edge) = Due to the method used to define arrays there is a gene at one edge of the region BUT the high concentration of LINEs falls in the intergenic region **(B) Imprinted gene clusters are not enriched for LINE dense arrays.** Percentage of regions covered by LINE arrays. Arrays were defined as >40% LINE content over >50Kb. Twenty-one imprinted clusters were analysed. The region was taken from the furthest points of the two known imprinted genes at the edges of the cluster. For singleton imprinted genes which lie within a host gene, the region was defined as the coordinates of the host gene. Flanking regions 1 Mb upstream and 1 Mb downstream of each imprinting cluster were analysed. For random clusters 504 size matched regions and the 1 Mb upstream and 1 Mb downstream of each region were analysed. Wilcoxon matched pair tests were performed on the imprinted clusters versus random clusters and imprinted flanking regions versus random flanking regions. No significant difference was seen.

Supplemental Fig S2

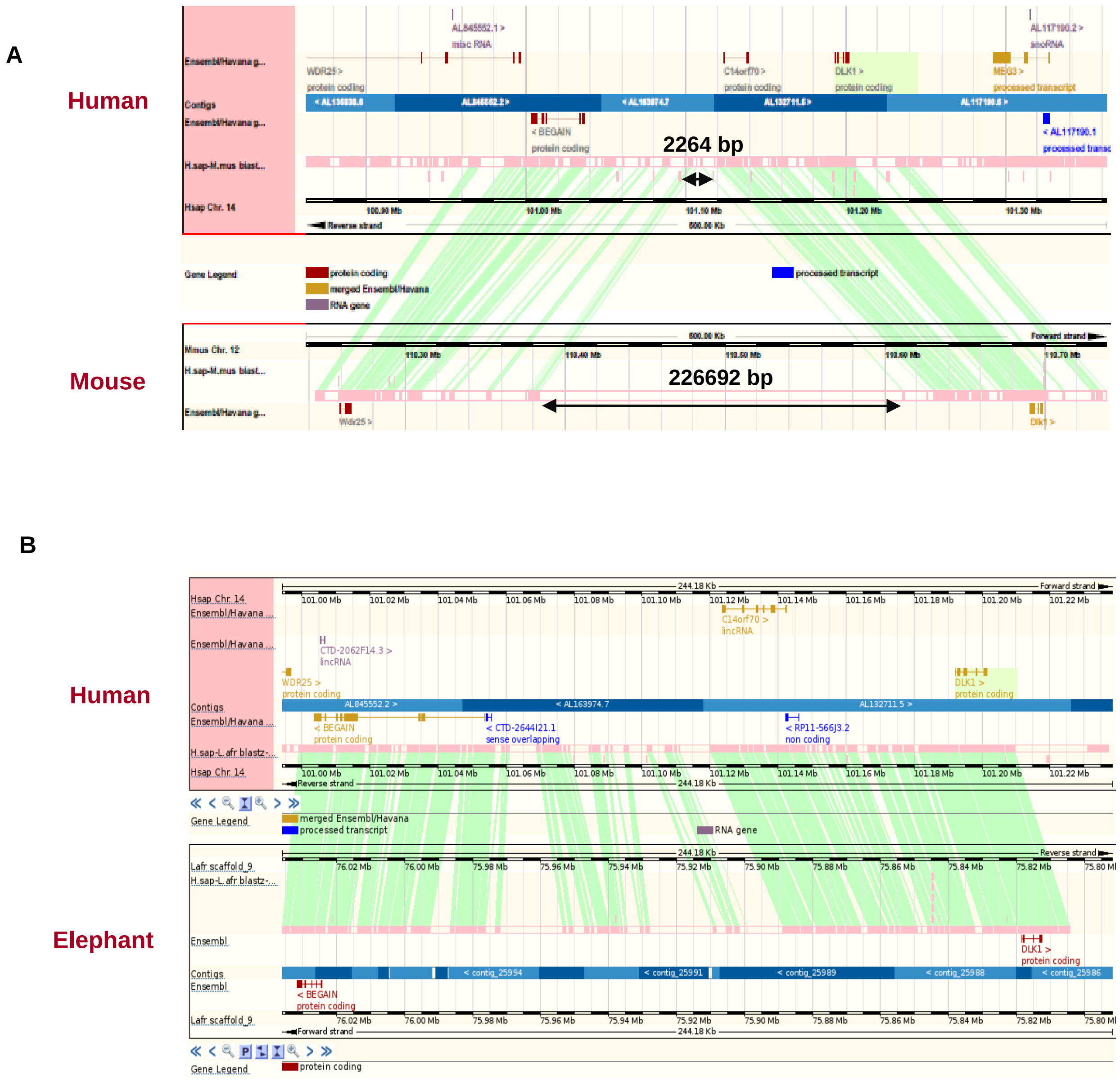

Supplemental Fig S2. Expansion in the mouse and rat *Begain-Dlk1* intergenic regions is due to the insertion of LINE-1s within a small interval. (A) Blastz alignments between the human *Begain-Dlk1*region and the orthologous sequence in mouse and rat. The inserted regions are indicated by double headed arrows. (B) Blastz alignments between elephant and human *Begain-Dlk1* region illustrating a more uniform expansion in the elephant. Images are taken from Multi-Species View in Ensembl (Flicek et al. 2011). Blastz alignments are shown in green and conserved elements are shown in pink.

Supplemental Fig S3

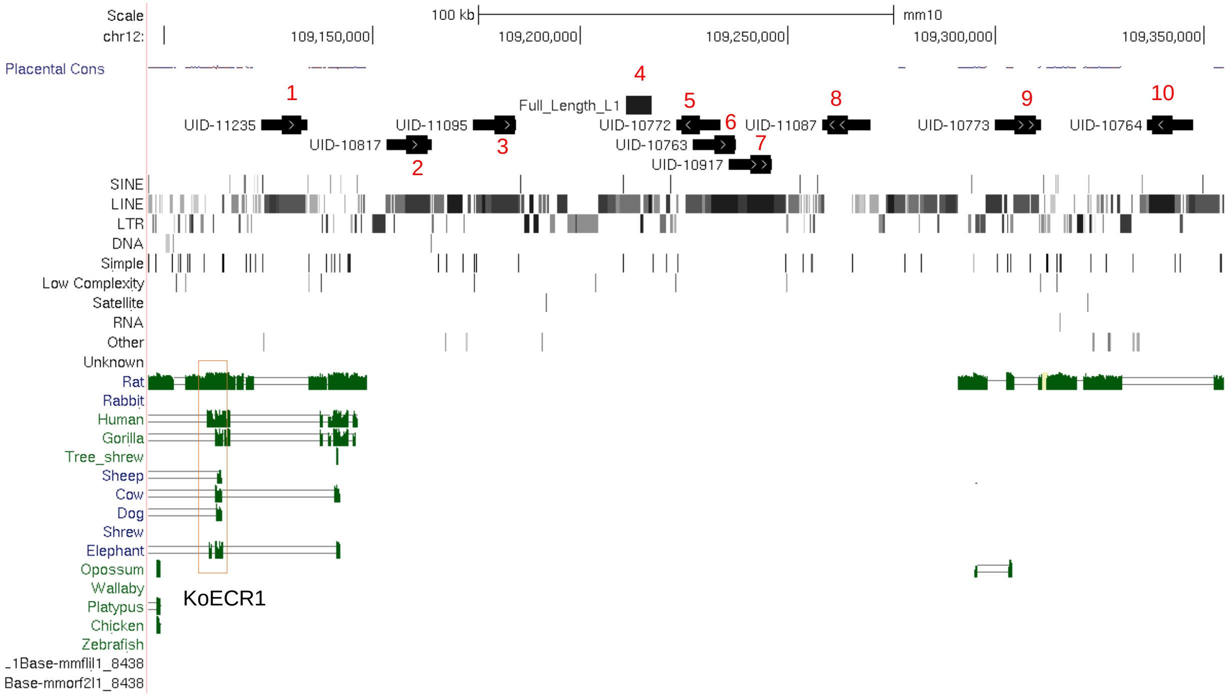

Supplemental Fig S3. Ten full length non-intact L1 elements are located in the *del*L1rep deletion. The nine full length non-intact L1s from L1Base2 (Penzkofer et al. 2016) are indicated by their database numbers. A further full length L1 was found using L1Xplorer (Penzkofer et al. 2005), and is labelled "Full-length L1". The numbers in red correspond to the IDs in Supplemental Table S4. The red box indicates the position of the putative enhancer (koECR1) located within the deleted interval. Image taken from UCSC Genome Browser (Kent et al. 2002) with the mm10 full length non-intact L1 bed file downloaded from L1Base2 (Penzkofer et al. 2016).

Supplemental Fig S4

A

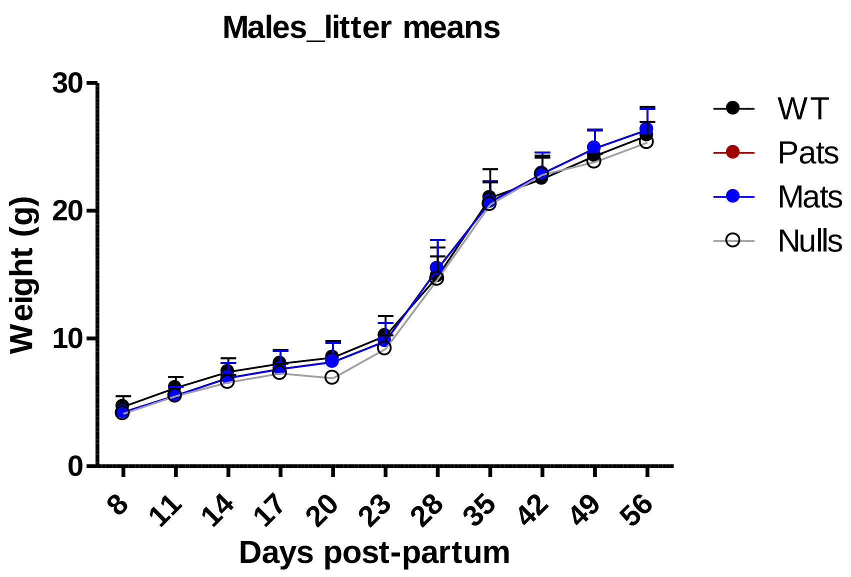

B

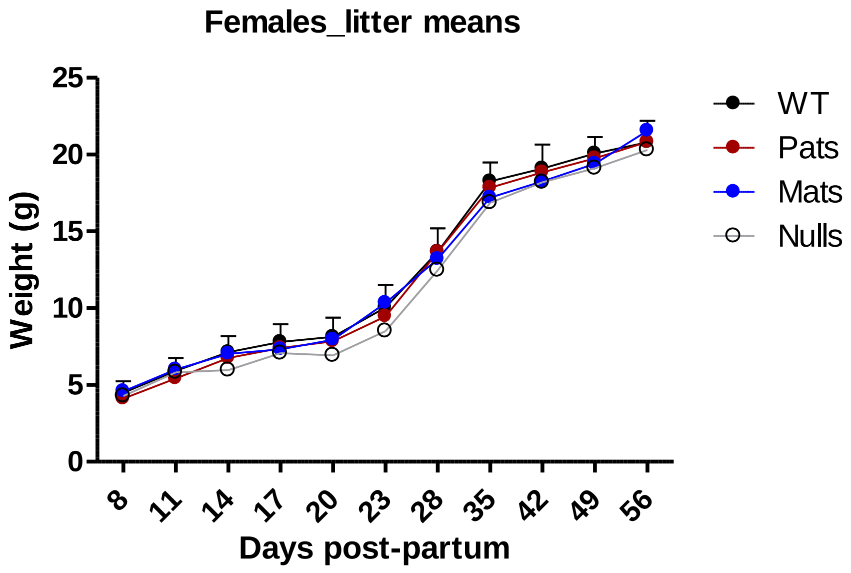

Supplemental Fig S4. No growth phenotype was observed upon transmission of *del*$^{\mathbf{L1rep}}$.

Growth curves of *del*^{L1rep} mutant mice. (A) male mutants and wild type littermates (wt n ≥19; paternal transmission n ≥7; maternal transmission n ≥7; null n ≥3). (B) female mutants and wild type littermates (wt n ≥13; paternal transmission n ≥7; maternal transmission n ≥2; null n ≥5). Data were compared by 2-way analysis of variance and no significant difference was seen.

Supplemental Fig S5

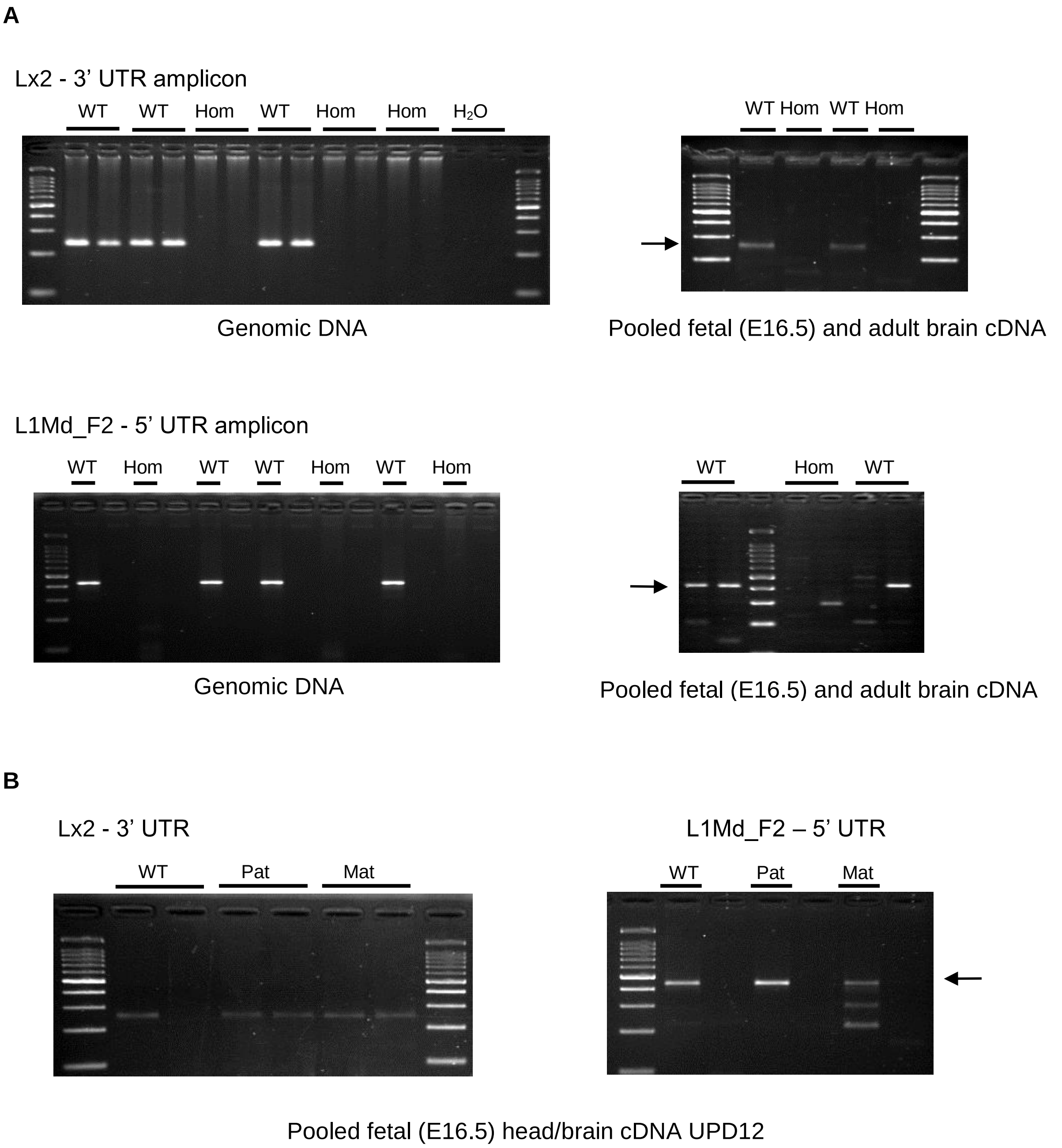

Supplemental Fig S5. LINE-1 elements from within the deleted repeat interval are transcribed but are not imprinted.

(A) - PCR amplification of Lx2-3'UTR and L1Md_F2-5'UTR sequence from genomic DNA and brain cDNA of wild type and del^{L1rep} homozygous mutant mice.

The absence of amplification from genomic DNA in homozygous mutants shows the amplicons are specific for the deleted genomic region. Left panels: samples were loaded in duplicate per genotype (Lx2-3'UTR) and in single interspersed lanes (L1Md_F2-5'UTR).

cDNA amplification in wild type mice demonstrated the existence of transcripts arising from these LINE elements, which are specific to the deleted interval as shown by lack of products in del^{L1rep} homozygous mutants. Right panels: samples were loaded in consecutive lanes (Lx2-3'UTR) and in duplicate per genotype (L1Md_F2-5'UTR). Amplification failed in one of the WT lanes (1st duplicate of #2); non-specific products were seen in its place, as well as in one of the del^{L1rep} homozygote lanes.

(B) PCR amplification of Lx2-3'UTR and L1Md_F2-5'UTR sequence from pooled head/brain cDNA of embryos with uniparental duplications of chromosome 12 (matUPD12 and patUPD12). Amplification was seen in both pat and matUPD12 embryos showing that transcription of these LINE-1 elements is not imprinted. Samples were loaded in duplicate per genotype (Lx2 - 3'UTR) and in single interspersed lanes (L1Md_F2-5'UTR). Non-specific products were seen in the matUPD12 sample, together with the expected product. Arrows denote the expected product size. Marker: 100 bp ladder.

Supplemental Fig S6

A *Dlk1*

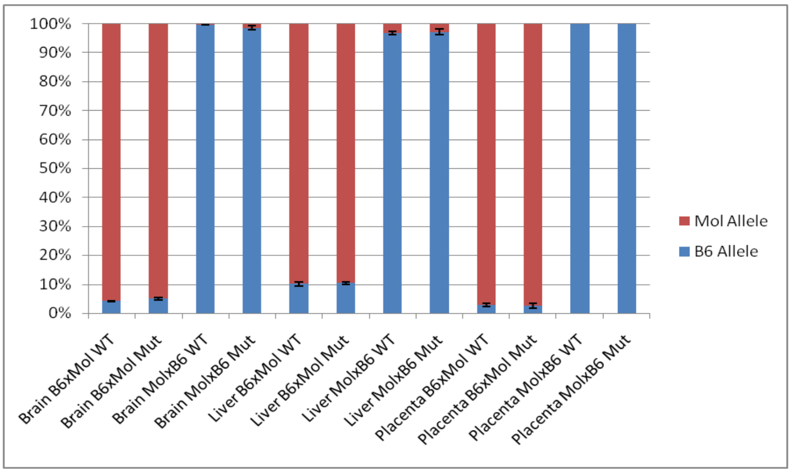

B *Gt12*

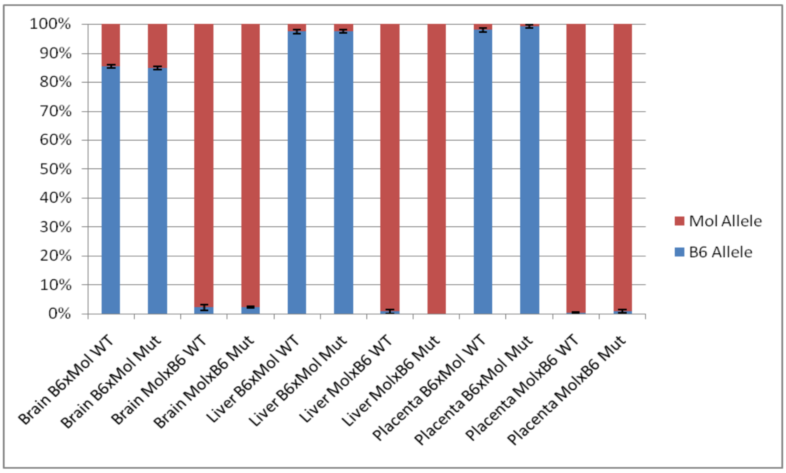

C *Dio3*

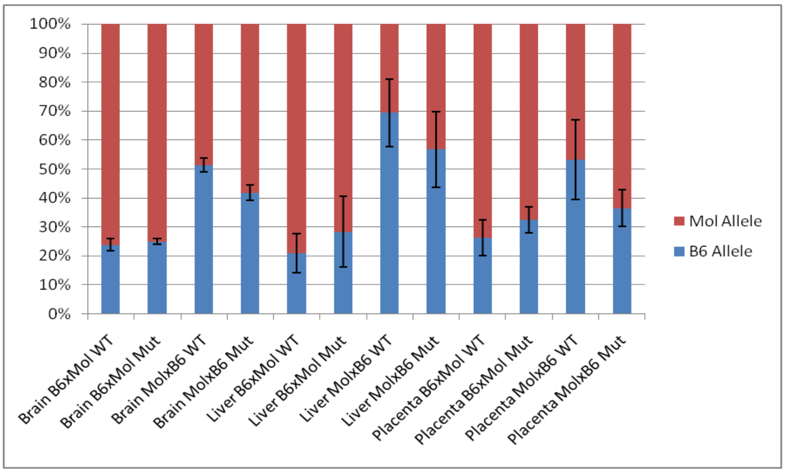

D *Rtl1as*

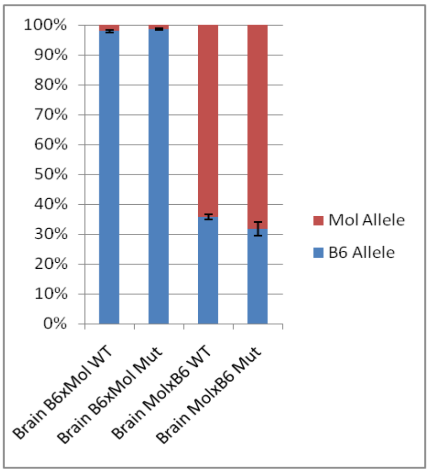

E *Begain* b

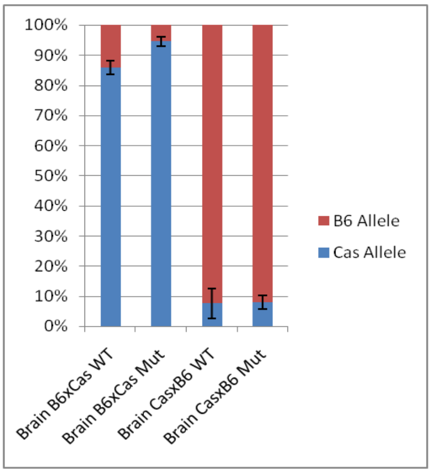

Supplemental Fig S6. The allele-specific status of the imprinted genes is unaffected by *del*^{L1rep}.

Allele-specific expression was quantitatively assessed using pyrosequencing. Wild type (WT) and heterozygous (Mut) embryos were obtained from reciprocal crosses between *del*^{L1rep} heterozygous mutants (C57BL/6 (B6) and Mus musculus molossinus 12 (Mol) for (a) *Dlk1*, (b) *Gtl2* (c) *Dio3* and (d*) Rtl1-as* or Mus musculus castaneus (CAST) for (e) *Begain* 1b. The graphs show percentage of each allele detected at the polymorphic base in cDNA generated from E16.5 brain liver or placenta. For each cross the maternal genotype is written first. The *del*^{L1rep}/B6 allele is shown in blue and the molossinus or castaneus allele is shown in red. Error bars represent the standard error of the mean.

Supplemental Fig S7

A *Wars*

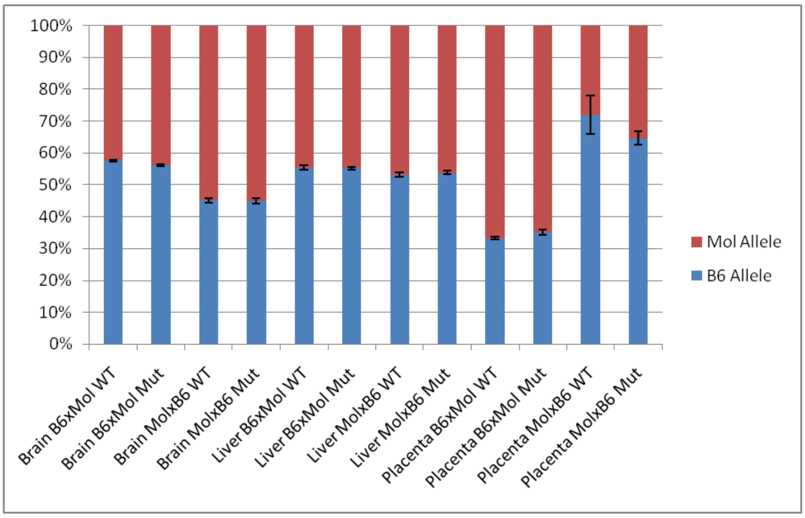

B *Wdr25*

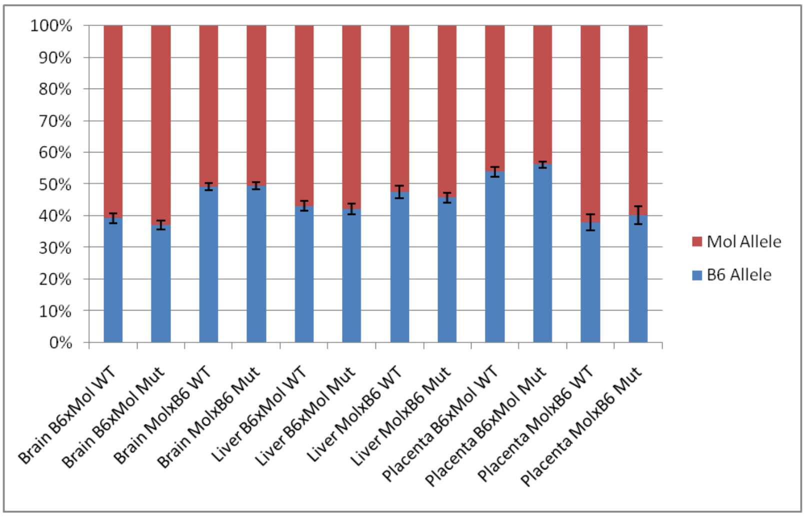

C *Begain* a

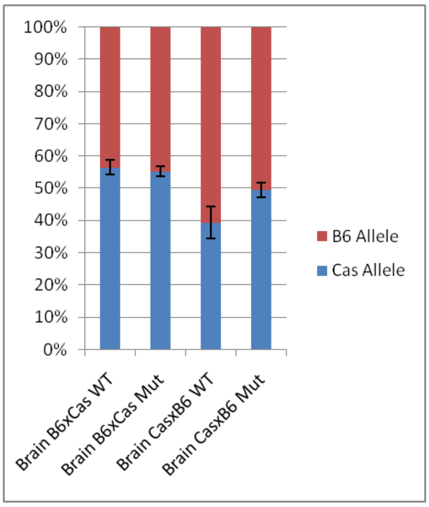

Supplemental Fig S7. Non-imprinted genes are unaffected by $\textit{del}^{\textbf{L1rep}}$.

Allele-specific expression was quantitatively assessed using pyrosequencing. Wild type (WT) and heterozygous (Mut) embryos were obtained from reciprocal crosses between $\textit{del}^{\mathrm{L1rep}}$ heterozygous mutants (C57BL/6 (B6) and Mus musculus molossinus 12 (Mol) for (a) *Wars* and (b) *Wdr25* or Mus musculus castaneus (CAST) for (c) *Begain* 1a. The graphs show percentage of each allele detected at the polymorphic base in cDNA generated from E16.5 brain liver or placenta. For each cross the maternal genotype is written first. The $\textit{del}^{\mathrm{L1rep}}$/B6 allele is shown in blue and the molossinus or castaneus allele is shown in red. Error bars represent the standard error of the mean.

Supplemental Fig S8

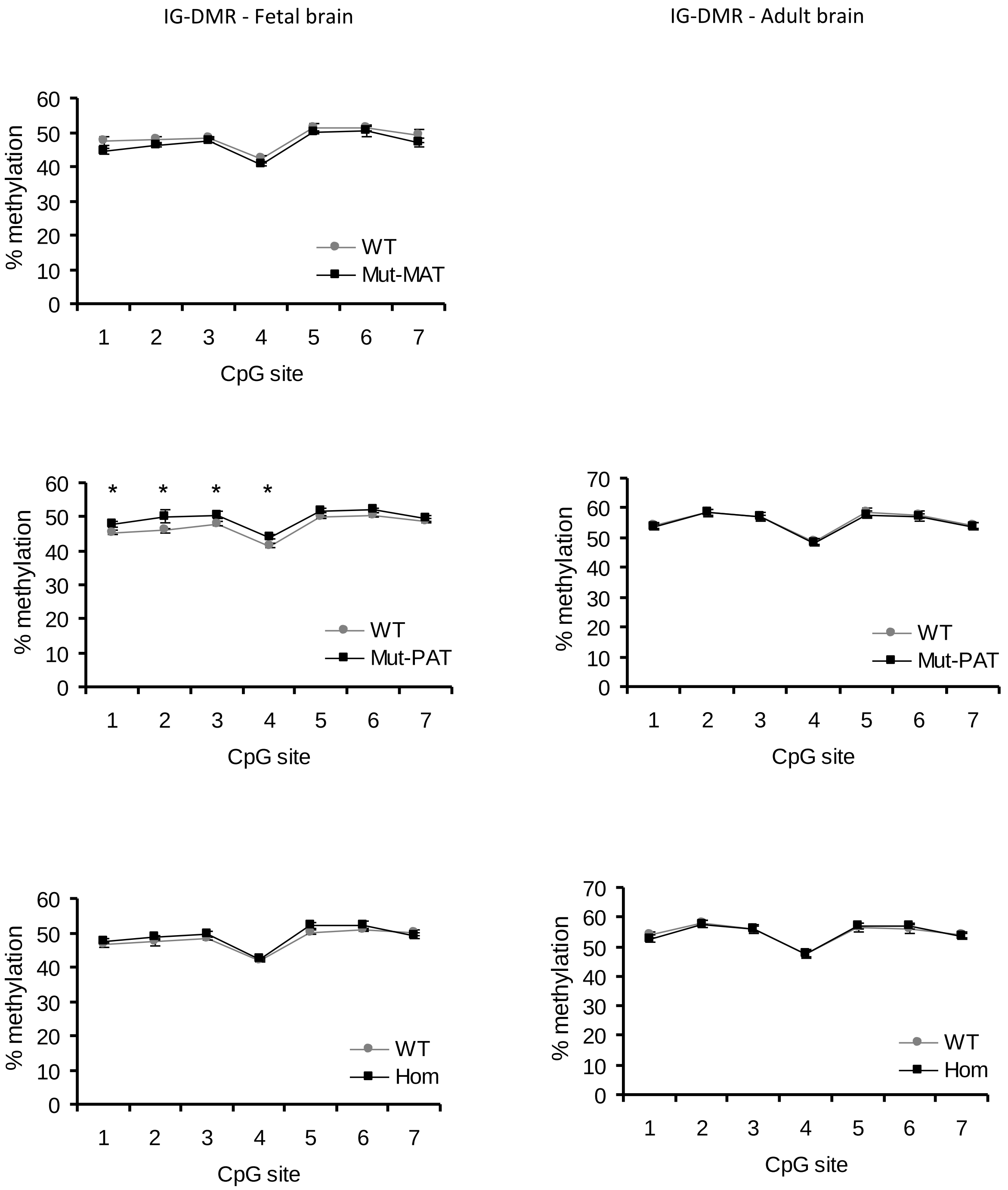

Supplemental Fig S8. *del*^{L1rep} does not impact on methylation of the IG-DMR.

Assessment of the methylation levels of the imprinting control centre IG-DMR by pyrosequencing in fetal and adult brain. Upper panels, maternal transmission of the deletion (per genotype n=5, 2 litters); middle panels, paternal transmission (fetal: per genotype n=9, 5 litters; adult: per genotype n=10, 5 litters); lower panels, homozygote intercrosses (per genotype n=6, 4 litters; adult: per genotype n=7, 5 litters).

The methylation profile of the IG-DMR was generally at the expected level of ~50% in control and mutant embryos. A negligible yet statistically significant increase in methylation is seen in 4 of the tested 7 CpG sites of the IG-DMR in fetal brain upon *del*^{L1rep} paternal inheritance (2.5%, 4.2%, 2.5% and 2.6%, respectively) (n= 9 wt, 9 mut; *$p < 0.05$); however these values fall within the margin of error of the allele-specific methylation methodology (3%) (Wong et al. 2006) and the increase is not recapitulated in fetal brain of homozygous mutants, nor is it seen in adult brain. Error bars denote SEM.

Supplemental Fig S9

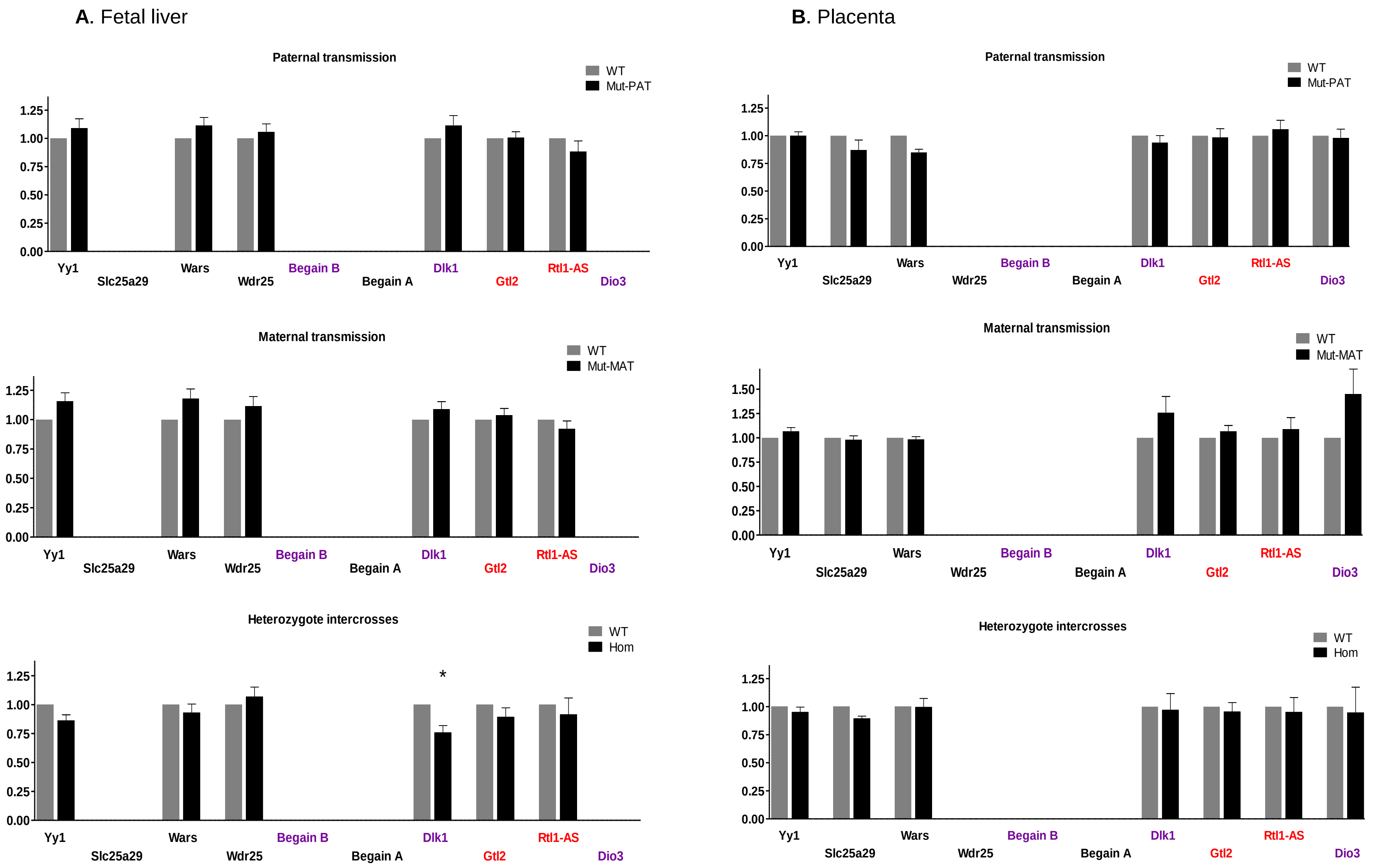

Supplemental Fig S9 - Gene expression in fetal liver and placenta is unaffected by *del*^{L1rep}.

Relative expression of five biallelic genes closest to the deleted interval (black) and of the transcripts within the neighbouring imprinted cluster (blue, paternally expressed; red, maternally expressed) in fetal (E16.5) liver (A) and placenta (B), as determined by RT-qPCR. No significant differences in expression were observed upon heterozygous or homozygous inheritance of the *del*^{L1rep} allele. Maternal transmission, n= 12 wt, 14 mut; 4 litters. Paternal transmission, n= 13 wt, 13 mut; 4 litters. Heterozygote intercrosses, n= 12 wt, 13 hom; 6 litters.

Slc25a29, *Begain* and *Dio3* are not expressed or only residually expressed in liver; *Wdr25* and *Begain* are not expressed or only residually expressed in placenta.

Data was normalized to *beta-2-microglobulin* expression and is shown relative to WT controls (= 1).*p < 0.05, **p < 0.01, ***p < 0.001 by two-tailed Student's t-test.

Supplemental Fig S10

Adult brain

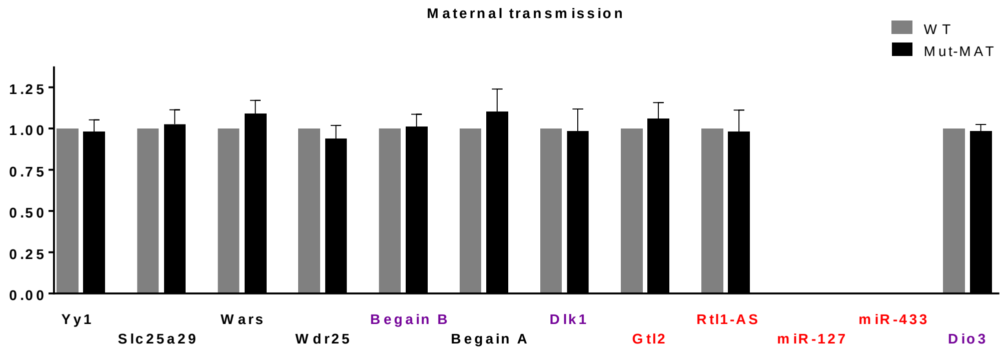

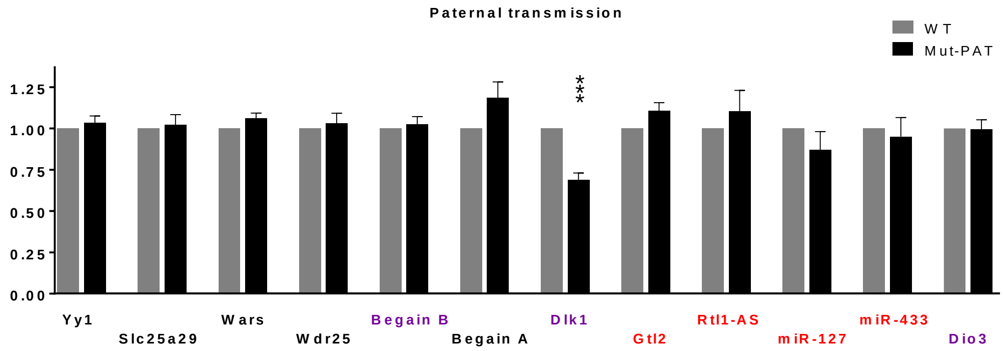

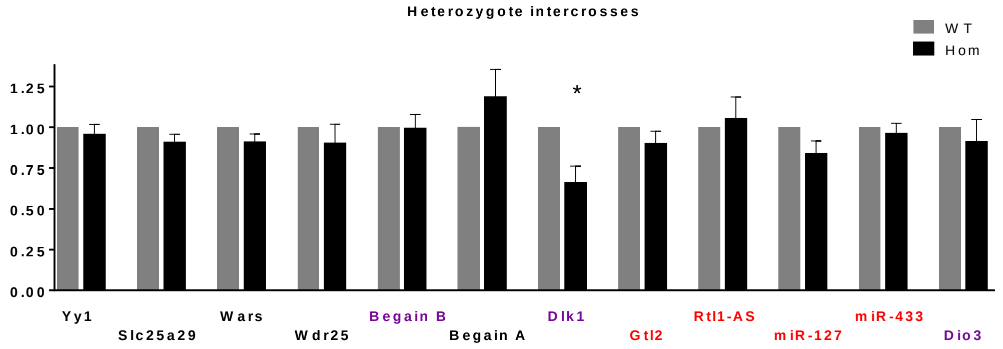

Supplemental Fig S10. Paternal transmission of *del*^{L1rep} elicits the down regulation of *Dlk1* in adult brain.

Relative expression of five biallelic genes closest to the deleted interval (black) and of the transcripts within the neighbouring imprinted cluster (blue, paternally expressed; red, maternally expressed) in adult brain, as determined by RT-qPCR. Gene expression in adult brain is mostly unaffected by delL1rep. No changes were observed upon maternal transmission of delL1rep (n= 3 wt, 3 mut; 2 litters). However, paternal inheritance of the mutant allele (upper panel) elicited a significant down regulation of Dlk1 (n= 10 wt, 10 mut; 5 litters), which was recapitulated in homozygous mutants (lower panel) (n= 7 wt, 7 mut; 5 litters).

Data was normalized toGap3dh expression and is shown relative to WT controls (= 1).*p < 0.05, **p < 0.01, ***p < 0.001 by two-tailed Student's t-test.

Supplemental Fig S11

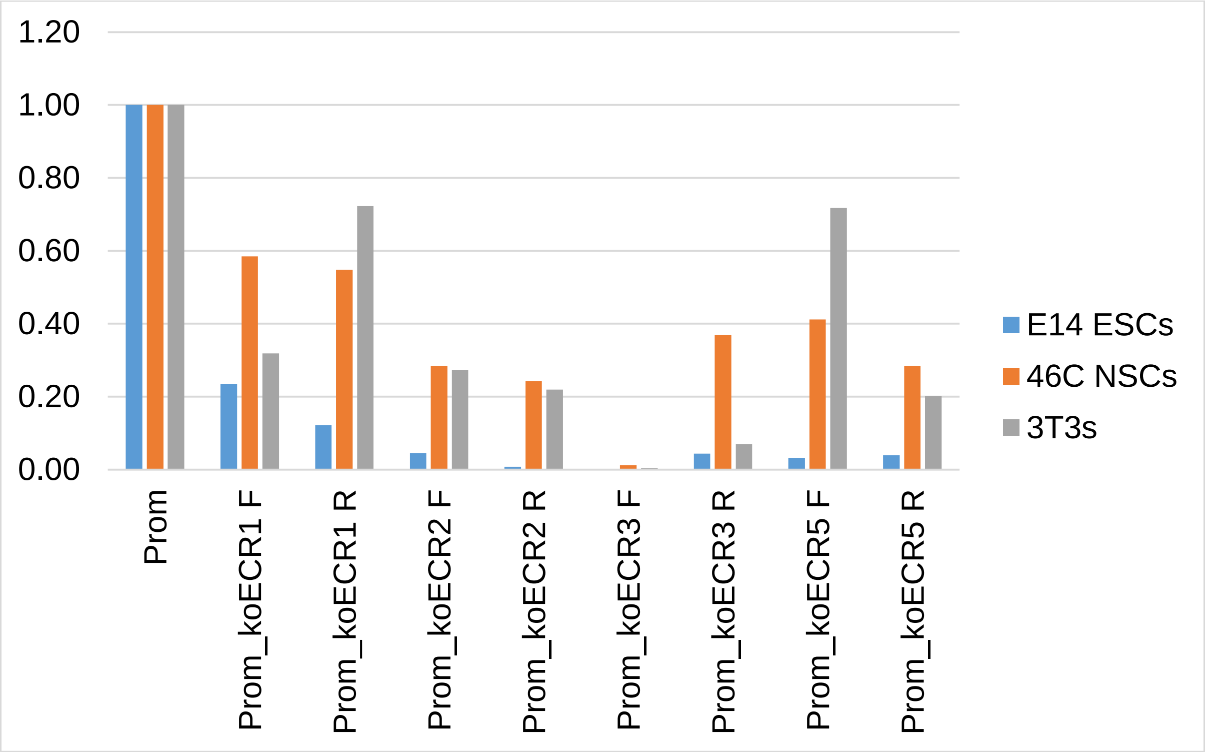

Supplemental Fig S11. ECRs in the *del*^{L1rep} knock-out region show no enhancer activity in luciferase reporter assays conducted in ESCs, NSCs or embryonic fibroblasts. koECRs 1, 2, 3, and 5 were cloned into pGL3-Promoter (Promega) in both orientations to test for enhancer activity. Constructs were transfected into murine ESCs (E14tg2a), neural stem cells derived from ESCs (46C NSCs) or mouse embryonic fibroblasts (NIH-3T3). Graph shows the expression as measured in luminescence of luciferase relative to renilla and normalised to the empty pGL3-Promoter vector (Prom).

Supplemental Table S1

chr	start	End	Gene content	Genes in region	LADS
chr1	97360001	97460000	Intergenic		fLAD
chr1	117790001	118010000	Intragenic and Flanking	B020011L13Rik, Gm28360, Gm28168, Gm7145	cLAD
chr1	118020001	118150000	Intergenic		cLAD
chr2	36540001	36640000	Flanking	Olfr344	cLAD
chr2	36890001	36990000	Flanking	Olfr353, Olfr354, Olfr355, Olfr356	cLAD
chr2	37220001	37330000	Intergenic (gene at edge)	Olfr366 (Olfr367-ps-pseudogene)	cLAD
chr2	89680001	89790000	Flanking	Olfr1252, Olfr1253, Olfr1254	cLAD
chr2	95240001	95350000	Intergenic		cLAD
chr2	111880001	111980000	Flanking	Olfr1306, Olfr1307, Olfr1308	cLAD
chr3	3390001	3490000	Intergenic		fLAD
chr3	4020001	4140000	Intergenic		fLAD
chr3	17350001	17460000	Intergenic		cLAD
chr3	90830001	91030000	Intergenic		cLAD
chr3	106580001	106760000	Intragenic and Flanking	Lrif1	cLAD
chr3	106830001	106940000	Intragenic and Flanking	RP24-481E4.4 Gm27008	cLAD
chr4	17370001	17470000	Intergenic		cLAD
chr4	37560001	37690000	Intergenic		cLAD
chr4	113960001	114080000	Intragenic and Flanking	Skint5	fLAD
chr4	114150001	114290000	Intragenic and Flanking	Skint11	fLAD
chr5	109300001	109400000	Intragenic and Flanking	Vmn2r16	cLAD
chr6	11360001	11470000	Intergenic		cLAD
chr6	42070001	42170000	Intragenic and Flanking	Tas2r139	cLAD
chr6	57850001	57950000	Flanking	Vmn1r22, Vmn1r23	fLAD
chr6	58330001	58440000	intergenic (gene at edge)	Vmn1r30	fLAD
chr6	122990001	123090000	Intragenic and Flanking	Clec4a4 and Clec4b1	cLAD
chr6	131080001	131190000	Intragenic and Flanking	Gm5581	cLAD
chr6	131730001	131830000	Intergenic		cLAD
chr7	3960001	4060000	Intragenic and Flanking	Lair2	fLAD
chr7	5120001	5250000	Flanking	Rasl2-9, Vmn1r55, Vmn1r56, Vmn1r57	cLAD
chr7	5500001	5650000	Flanking	Vmn1r60, Vmn1r61	cLAD
chr7	41750001	41980000	Intragenic and Flanking	Vmn2r58	cLAD
chr7	42130001	42260000	Intragenic and Flanking	Vmn2r60	cLAD
chr7	42270001	42390000	Intragenic and Flanking	Vmn2r61	cLAD
chr7	48620001	48720000	Flanking	Mrgprb3	fLAD
chr7	62460001	62620000	Intergenic (gene at edge)	Peg12 (Atp5l-ps1-pseudogene)	cLAD
chr7	62720001	62930000	Intergenic		cLAD
chr9	3650001	3750000	Intragenic	Gucy1a2	fLAD
chr9	36540001	36660000	Flanking	Gm5615, Gm17689, A63009E13Rik	cLAD
chr10	47850001	47960000	Intergenic		fLAD
chr10	52550001	52670000	Intergenic		fLAD
chr11	11280001	11390000	Intragenic	Zpbp	fLAD
chr12	109150001	109320000	Intergenic		fLAD
chr14	14190001	14290000	Intergenic		cLAD
chr14	50490001	50660000	Flanking	Olfr742, Olfr743, Olfr744, Olfr745, Olfr746	cLAD
chr14	124690001	124800000	Intergenic		interLAD
chr15	33700001	33810000	Intergenic		cLAD
chr17	38810001	38990000	Intergenic		fLAD
chr18	90230001	90480000	Intergenic		fLAD
chr19	9680001	9830000	Intergenic		cLAD
chr19	39110001	39250000	Intragenic	Cyp2c66	fLAD
chrX	5500001	5670000	Intergenic (gene at edge)	Gm14374	interLAD
chrX	39240001	39380000	Flanking	Cypt15	cLAD
chrX	49500001	49620000	Intergenic (gene at edge)	Arhgap36	cLAD
chrX	50010001	50330000	Intergenic (gene at edge)	Olfr1323	cLAD
chrX	80340001	80440000	Intergenic		cLAD
chrX	97450001	97630000	Intergenic		fLAD
chrX	97790001	97980000	Intergenic		fLAD
chrX	105520001	105650000	Intergenic		cLAD
chrX	107950001	108060000	Intergenic		fLAD
chrX	110010001	110140000	Intergenic		fLAD
chrX	121100001	121200000	Intergenic		fLAD
chrX	126250001	126350000	Intergenic		fLAD
chrX	132560001	132670000	Intergenic		cLAD
chrX	150690001	150800000	Intergenic		fLAD
chrX	155950001	156080000	Intragenic	Gm15155	cLAD
chrY	1470001	1710000	Intergenic		N/A*

Supplemental Table S1. The 66 mouse LINE rich arrays as defined by >70% LINE content over >100 Kb. Genomic contexts are defined as follows: Intragenic = LINE array falls within a gene, Flanking = LINE array surrounds small genes in the region, Flanking & Intragenic = LINE array surrounds and is located within genes in the region, Intergenic = LINE array falls between genes, Intergenic (gene at edge) = Due to method used to define arrays there is a gene at one edge of the region BUT the high concentration of LINEs falls in the intergenic region. Overlapping LADs are classified as: fLAD = facultative LAD, cLAD = constitutive LAD (as defined by Peric-Hupkes et al., 2010). LAD data is not available for chromosome Y. Coordinates are for mouse genome build mm10.

Supplemental Table S2

	Human (hg38)	Rhesus (rheMac3)	Marmoset (calJac3)	Mouse (mm10)	Rat (rn5)	Guinea pig (cavPor3)	Panda (ailMel1)	Elephant (loxAfr3)	Opossum (monDom5)
***Begain - Dlk1* region**	Chr14:100567924-100727069	Chr7:164465840-164621893	Chr10:126489588-126622138	Chr12:109068167-109453659	Chr6:142128557-142742149	Scaffold_111:189820-799867	GL192537.1:469660-612737	Scaffold_9:75820098-76004764	Chr1:317498851-318030058
**Size**	159146 bp	156054 bp	132551 bp	385493 bp	613593 bp	610048 bp	143078 bp	184667 bp	531208 bp
**Repeat content (%)**									
**SINEs**	12.11	11.93	11.34	3.42	1.64	1.44	6.48	10.11	15.50
**LINEs**	12.05	11.72	9.62	48.52	34.35	63.18	14.25	21.20	35.16
**LTR elements**	10.15	10.33	8.24	15.34	8.42	3.19	5.87	11.89	10.58
**DNA elements**	2.04	2.01	1.98	1.00	0.41	0.20	1.66	1.51	2.09
**Unclassified**	0.00	0.00	0.00	1.16	1.92	0.00	0.00	0.00	0.00
**Small RNA**	0.00	0.00	0.00	0.04	0.08	0.05	2.08	0.23	0.13
**Simple Repeats**	1.76	1.40	1.49	2.30	1.22	0.41	2.02	1.48	1.45
**Low complexity**	0.14	0.20	0.20	0.20	0.26	0.07	0.36	0.08	0.14
**Gaps in assembly**	0.00	1.93	8.00	0.00	34.98	11.10	1.45	2.14	0.44
**Unmasked**	61.75	60.48	59.12	28.02	16.75	20.37	65.83	51.37	34.51
**Genome Content Summary (%)**									
**SINEs**	13.47	13.59	13.64	7.70	7.62	5.70	8.60	10.30	11.79
**LINEs**	21.78	20.10	22.60	20.32	19.03	22.01	21.48	35.83	30.10
**LTR elements**	9.19	9.38	7.56	12.00	10.27	7.42	5.50	7.50	9.90
**DNA elements**	3.62	3.86	3.35	1.09	1.11	1.70	3.28	2.51	2.32
**Simple Repeats**	1.53	1.43	1.37	3.05	2.91	1.34	1.32	0.84	2.05
**Other**	2.55	0.55	0.00	0.50	1.38	0.00	0.00	0.40	0.00
**Unclassified**	0.33	0.35	0.51	0.33	0.23	0.38	0.45	0.23	0.31
**Unmasked**	47.52	50.73	50.95	55.01	57.46	61.45	59.37	42.38	43.52

Supplemental Table S2. Size and repeat content of Begain to Dlk1 in nine different species. The repeat content was ascertained using RepeatMasker (Smit et al. 2015) and the whole genome repeat content was taken from the RepeatMasker Genomic Datasets. There appears to be an inversion involving *Begain* and part of the neighbouring *Wdr25* gene in the rat, therefore in this species the region is defined as *Wdr25* to *Dlk1*.

Supplemental Table S3

	Pre-masked repeats			UCSC Repeats		
	Begain-Dlk1	KO region	Insertion	Begain-Dlk1	KO region	Insertion
**Number of LINEs**	237	180	157	231	178	157
**Number of L1s**	231	179	157	226	177	157
**Number of L1Mds**	47	44	40	21	19	19
**L1Md_A**	10	8	8	0	0	0
**L1Md_Gf**	4	4	4	0	0	0
**L1Md_Tf**	2	1	1	0	0	0
**Number Mammalian**	1	0	0	1	0	0
**Number Therian**	4	1	0	3	1	0
**Number Eutherian**	34	13	12	32	13	13
**Number Euarchontoglires**	8	7	4	8	7	4
**Number Rodentia**	54	34	24	54	35	25
**Number Muridae**	86	78	74	91	82	76
**Number Mus**	50	47	43	42	40	39
**% Mammalian**	0.4	0.0	0.0	0.4	0.0	0.0
**% Therian**	1.7	0.6	0.0	1.3	0.6	0.0
**% Eutherian**	14.3	7.2	7.6	13.9	7.3	8.3
**% Euarchontoglires**	3.4	3.9	2.5	3.5	3.9	2.5
**% Rodentia**	22.8	18.9	15.3	23.4	19.7	15.9
**% Muridae**	36.3	43.3	47.1	39.4	46.1	48.4
**% Mus**	21.1	26.1	27.4	18.2	22.5	24.8
**% Rodent specific**	80.2	88.3	89.8	81.0	88.2	89.2
**% Muridae specific**	57.4	69.4	74.5	57.6	68.5	73.2
**% Mds**	19.8	24.4	25.5	9.1	10.7	12.1
**L1Md_A**	4.2	4.4	5.1	0.0	0.0	0.0
**L1Md_Gf**	1.7	2.2	2.5	0.0	0.0	0.0
**L1Md_Tf**	0.8	0.6	0.6	0.0	0.0	0.0

Supplemental Table S3. Analysis of the LINE-1 content in the *Begain-Dlk1* intergenic region in mouse. KO region is the region deleted in this study. Insertion is the region between the two ECRs that flank the repeat array. Information on the lineage specificity of the LINE repeats was obtained from RepeatMasker (http://www.repeatmasker.org/ (Smit et al. 2015)) and Repbase (http://www.girinst.org/repbase/). Pre-masked repeats were downloaded from RepeatMasker and the UCSC repeat masker track was downloaded from UCSC Tablemaker (Kent et al. 2002; Karolchik et al. 2004).

Supplemental Table S4

ID	L1Base2_ID	Chromosome	Start	End	Strand	ORF1 gaps	ORF1 frameshifts	ORF1 stops	ORF2 gaps	ORF2 frameshifts	ORF2 stops	PolyA Signal	Annotation	Used for expression analysis
1	11235	12	109123362	109134462	+	4	1	4	97	13	15	cons	Lx2	✓
2	10817	12	109153490	109164430	+	0	1	4	4	6	4	mut	L1_Mus2	
3	11095	12	109174101	109184472	+	0	2	1	0	7	5	mut	L1Md_F2	
4	not in L1Base2	12	109211066	109217193	+	0	3	2	20	16	28	cons	L1VL2	
5	10772	12	109233900	109223139	-	3	1	2	191	15	11	mut	Lx	
6	10763	12	109227134	109237403	+	2	3	0	2	4	3	mut	L1Md_F3	
7	10917	12	109235833	109246126	+	0	1	0	0	5	0	mut	L1Md_T	
8	11087	12	109269798	109258354	-	0	1	1	2	6	6	mut	L1Md_F2	✓
9	10773	12	109299717	109310808	+	5	5	2	7	15	17	cons	Lx	
10	10764	12	109347519	109336342	-	0	1	2	4	6	6	cons	L1Md_F3	

Supplemental Table S4 – Full length, non-intact L1 elements within the *del*$^{\mathbf{L1rep}}$ deleted region. Nine full length non-intact L1s were identified in L1Base2 (Penzkofer et al. 2016) and a further full length L1 was found using L1Xplorer (Penzkofer et al. 2005). Co-ordinates are for mm10. Columns indicate the number of gaps, frameshifts and stops within the ORF1 and ORF2 sequences which render them incapable of retrotransposition. Annotation is taken from RepeatMasker (Smit et al. 2015). Mut: mutated; Cons: conserved. The two elements used for subsequent expression analysis are indicated in final column.

Supplemental Table S5

Viability & Reproductive Fitness							
**Pups**	**Maternal transmission**		**Paternal transmission**		**Heterozygote intercrosses**		
	+ / +	***del*^{L1rep}/+**	**+ / +**	**+/*del*^{L1rep}**	+ / +	**+ / -**	**- / -**
No. of pups born	89	83	98	109	60	105	53
No. litters	24		32		28		
Average litter size	7.2		6.7		7.6		
No. of pups at wean	76	68	73	82	44	84	45
No. of dead pups	13	15	25	27	16	21	8
Avg litter size at wean	5.9		5		6.3		
No. of male pups	38	38	40	41	17	49	17
No. of female pups	37	26	33	41	27	35	28
Ratio Male : Female	1:1	1.2 : 0.8	1.1 : 0.9	1:1	0.8 : 1.2	1.2 : 0.8	0.8 : 1.2
Ratio wt: mut / wt: het: hom	1.0 : 1.0		0.9 : 1.1		1.1 : 1.9 : 1.0		
**Embryos (E16.5)**	**Maternal transmission**		**Paternal transmission**		**Heterozygote intercrosses**		
	+ / +	***del*^{L1rep}/+**	**+ / +**	**+/*del*^{L1rep}**	+ / +	**+ / -**	**- / -**
No. of embryos	22	20	19	16	20	50	21
No. litters	5		5		12		
Average litter size	8.6		7.0		7.6		
Ratio wt: mut / wt: het: hom	1.0 : 1.0		1.1 : 0.9		0.9 : 2.2 : 0.9		

Survival Analysis							
	**Maternal transmission**		**Paternal transmission**		**Heterozygote intercrosses**		
	+ / +	***del*^{L1rep}/+**	**+ / +**	**+/*del*^{L1rep}**	+ / +	**+ / -**	**- / -**
No. monitored	5	10	6	10	-	-	8
No. reaching 57 wks	4	10	6	9	-	-	8
Survival rate	80%	100%	100%	90%	-	-	100%

Supplemental Table S5. Viability, reproductive fitness and survival after maternal or paternal transmission of the mutant allele and heterozygote intercrosses.

Supplemental Table S6

	WT	PAT	MAT	NULL
**Number of values**	25	6	9	8
**Mean**	0.518	0.313	0.559	0.355
**Std. Deviation**	0.109	0.0665	0.094	0.214
**Std. Error**	0.0219	0.0272	0.0313	0.0756

Supplemental Table S6. Numbers of samples and data used in ELISA experiments shown in Figure 3b.

Supplemental Table S7

Transcript	Primers	Annealing (ºC)	Cycle No.	SNP
Wars	F: AGGGGCTGAAGGACGTCTA<br>R: [biotin] TCCTTGAAGATGACGACAGG<br>Seq: CTTCTTGAGCTCCCC	60	30 - Br<br>31 - Lv<br>30 - Pl	rs13474808 (Exon 11)<br>B6 /Mol
Wdr25	F: [biotin] GCTGCCTGTCTTAAGCCACTAAAA<br>R: CGGGGGCTCTCCATCTTT<br>Seq: CACCTGCTTCTGGATT	60	31 - Br<br>33 - Lv<br>33 - Pl	rs36938981 (Exon 2)<br>B6 /Mol
Begain v1A	F: GTAGTGGCGCGTGGAGTCAA<br>R: [biotin] GCAGAGCCGGGGCCATGT<br>Seq: GAGTCAAACTCCGTCT	60	32 - Br	rs36806417 (Exon 3)<br>B6 /Cast
Begain v1B	F: GTAGTGGCGCGTGGAGTC<br>R: [biotin] ATGGGCAGCCATCAGTCTT<br>Seq: GAGTCAAACTCCGTCTC	65	37 - Br	rs36806417 (Exon 3)<br>B6 /Cast
Dlk1	F: [biotin] CGCAAGAAGAAGAACCTCCTGT<br>R: ACGCTGCTTAGATCTCCTCATCA<br>Seq: CAGCCTCCTTGTTGAA	60	32 - Br	rs50424874 (Exon 5)<br>B6 /Mol
Gtl2	F: [biotin] CCCAGGACCCTCCAACTGTAAA<br>R: GTCAGCGCAGTTCATCAGTCA<br>Seq: GCGTCCCCGTGGCTG	60	32 - Br<br>34 - Lv<br>34 - Pl	rs46969056 (Exon 9)<br>B6 /Mol
Rtl1 AS	F: [biotin] ATGCCTCACTGAGTGGTTCC<br>R: TGTCAGGCAACCGTATTCACC (Mp-F)<br>Seq: GACTCCTGGTTCGAATT	65	28 - Br<br>28 - Lv<br>27 - Pl	chr12:110,832,593<br>NCBIm37<br>B6 /Mol
Dio3	F: GATGACGAACCGCCTCTAAC<br>R: [biotin] GTCTCGAAGTCCATCCCTTACC<br>Seq: TCCACAGGGAACCGT	60	35 - Br<br>39 - Lv<br>37 - Pl	chr12:111,519,018<br>NCBIm37<br>B6 /Mol

Supplemental Table S7 Primer sequences and amplification conditions for SNP-based allele-specific expression analyses by pyrosequencing. F: forward primer; R: reverse primer; Seq: sequencing primer. Br, brain; Lv, liver; Pl, placenta.

Supplemental Table S8

**Transcript**	**Primers**	**Annealing (ºC)**	**Note**
YY1	F: GCAAAGCGTTCGTTGAGAG<br>R: CGCAAATTGAAGTCCAGTGA	60	
Slc25a29	F: TGAGGCTTCTCTGTGCTCTG<br>R: CTCGACTTCCTGGCTGGAT	65	200 nM primer
Wars	F: AGGGGCTGAAGGACGTCTA<br>R: TCCTTGAAGATGACGACAGG	60	
Wdr25	F: ACAGCATTCAGTGGTGTCCA<br>R: CAGTGTCCCGAGTCCACAG	60	
Begain v1A	F: GTAGTGGCGCGTGGAGTC<br>R: GCAGAGCCGGGGCCATGT	68	200 nM primer
Begain v1B	F: GTAGTGGCGCGTGGAGTC<br>R: ATGGGCAGCCATCAGTCTT	65	200 nM primer
Dlk1	F: GAAAGGACTGCCAGCACAAG<br>R: CACAGAAGTTGCCTGAGAAGC	60	
Gtl2	F: GGACACACGGACACAGACA<br>R: TGTCCCACAGGAAATGTGCAA	60	
Rtl1-AS	F: ATGCCTCACTGAGTGGTTCC<br>R: *TGTCAGGCAACCGTATTCACC (Mp-F)*	69	200 nM primer
Dio3	F: CGCTGCTTCGGCAAAGCGCGA<br>R: GTCTCGAAGTCCATCCCTTACC	60	
Gapdh	F: CCATCACCATCTTCCAGGAG<br>R: GCATGGACTGTGGTCATGAG	60	
Beta 2-microglobulin	F: TCACCCCCACTGAGACTGATAC<br>R: CCAGTATGGCCGAGCCCA	60	

Supplemental Table S8 Primer sequences and annealing temperatures for quantitative PCR. F: forward primer; R: reverse primer. Denaturing at 95 ºC for 5 min and cycling at 95 ºC for 30 sec, annealing temperature for 60 sec.

Supplemental Table S9

L1 element	Primers	Annealing (ºC)	Size
**Lx2**-3'UTR (11235)	F: TGGATACCAGGCAAGTGAGA<br>R: TGGTCTCTGGATGCCCTTAA	65	241
**L1Md_F2**-5'UTR (11087)	F: AGACTACTTTCTACGGAGTCCTGA<br>R: GGAGGTCTGCAGGTGTGAAT	66	417

Supplemental Table S9 Primer sequences and annealing temperatures for amplification of Chr 12-specific LINE-1s from the deleted repeat array. F: forward primer; R: reverse primer

Supplemental Table S10

Name	Primer	Annealing (ºC)
KOECR1SacF<br>KOECR1SacR	5'-GGCCGAGCTCCGAGAAGGCAGCAGACAGAG<br>5'-GGCCGAGCTCCTGCCGCAGGGAGCTATT	65
KOECR2SacF<br>KOECR2SacR	5'-GGCCGAGCTCGGTTGCTGGATGTACCACCT<br>5'-GGCCGAGCTCTCCCCCAAAGACAACAGAAC	60
KOECR3NheF<br>KOECR3NheR	5'-GGCCGCTAGCGCTCCACAGGAGGAACAGAG<br>5'-GGCCGCTAGCTTCCATCAATCCATCCCTACA	60
KOECR5SacF<br>KOECR5SacR	5'-GGCCGAGCTCGATTCCCCTGTCCCTGTCTT<br>5'-GGCCGAGCTCAGTGGCCTTCCCTCAGATTT	60

Supplemental Table S10 Primer sequences and annealing temperatures for cloning ECRs within the *del*^{L1rep} Deleted region. Sac indicates that amplicon needs to be digested with SacI for subsequent cloning and Nhe indicates that amplicon needs to be digested with NheI. F: forward primer; R: reverse primer.

Supplemental References

Flicek P, Amode MR, Barrell D, Beal K, Brent S, Chen Y, Clapham P, Coates G, Fairley S, Fitzgerald S, et al. 2011. Ensembl 2011. *Nucleic Acids Res*. **39**:D800-6.

Karolchik D, Hinrichs AS, Furey TS, Roskin KM, Sugnet CW, Haussler D, Kent WJ. 2004. The UCSC Table Browser data retrieval tool. *Nucleic Acids Res* **32**: D493-6.

Kent WJ, Sugnet CW, Furey TS, Roskin KM, Pringle TH, Zahler AM, Haussler D. 2002. The human genome browser at UCSC. *Genome Res* **12**: 996–1006.

Smit AFA, Hubley R, Green P. 2015. RepeatMasker Open-4.0. http://www.repeatmasker.org.
